# Supplementary material for: A cAMP/PKA/Kinesin-1 Axis Promotes the Axonal Transport of Mitochondria in Aging Drosophila Neurons
Source: Curr Biol. 2018 Apr 23;28(8):1265–1272.e4. doi: 10.1016/j.cub.2018.02.048 (PMC5912900; doi:10.1016/j.cub.2018.02.048)
Supplement: Document S1. Figures S1–S3 and Tables S1 and S2 [file mmc1.pdf]

Current Biology, Volume *28*

Supplemental Information

A cAMP/PKA/Kinesin-1 Axis Promotes the Axonal Transport of Mitochondria in Aging *Drosophila* Neurons

Alessio Vagnoni and Simon L. Bullock

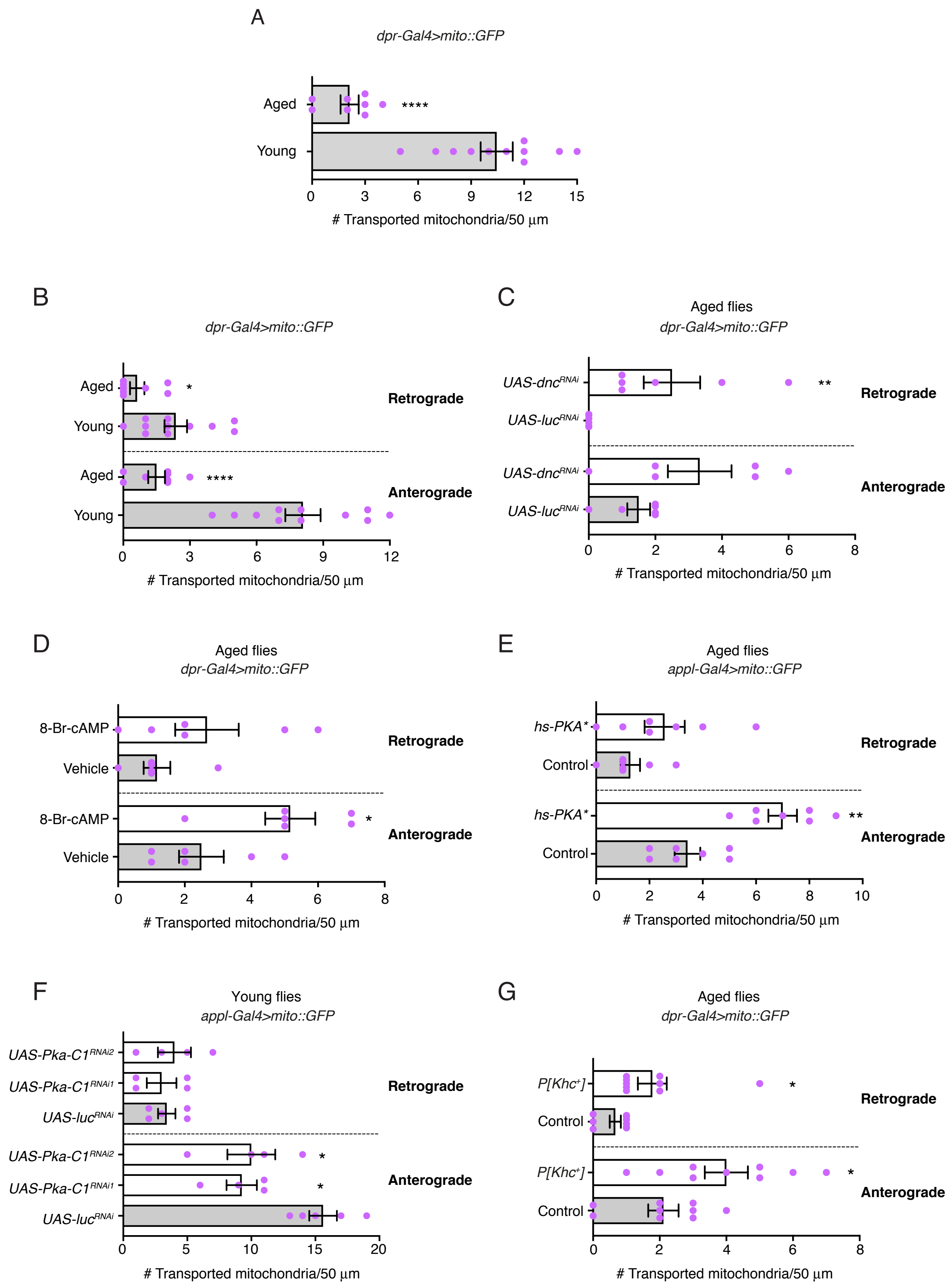

A
dpr-Gal4>mito::GFP
Aged
Young
****
0 3 6 9 12 15
Transported mitochondria/50 μm
B
dpr-Gal4>mito::GFP
Aged
Young
*
****
Retrograde
Anterograde
0 3 6 9 12
Transported mitochondria/50 μm
C
Aged flies
dpr-Gal4>mito::GFP
UAS-dnc RNAi
UAS-luc RNAi
**
Retrograde
Anterograde
0 2 4 6 8
Transported mitochondria/50 μm
D
Aged flies
dpr-Gal4>mito::GFP
8-Br-cAMP
Vehicle
*
Retrograde
Anterograde
0 2 4 6 8
Transported mitochondria/50 μm
E
Aged flies
appl-Gal4>mito::GFP
hs-PKA*
Control
**
Retrograde
Anterograde
0 2 4 6 8 10
Transported mitochondria/50 μm
F
Young flies
appl-Gal4>mito::GFP
UAS-Pka-C1 RNAi2
UAS-Pka-C1 RNAi1
UAS-luc RNAi
*
*
Retrograde
Anterograde
0 5 10 15 20
Transported mitochondria/50 μm
G
Aged flies
dpr-Gal4>mito::GFP
P[Khc+]
Control
*
*
Retrograde
Anterograde
0 2 4 6 8
Transported mitochondria/50 μm

Figure S1. Supplemental data for mitochondrial movements in wing neurons. Related to Figures 1, 2 and 4.

(A) Example of magnitude of reduction in mitochondrial transport in wing neurons of aged flies vs young flies. In these and other panels, mitochondria were marked with mito::GFP expressed in wing neurons using the Gal4-UAS system.

(B-G) Charts show number of transported mitochondria in the retrograde and anterograde direction per 50 μm of axonal tract per 3 min of image aquisition. Data in A and B derive from the same movies. Young and aged flies in A and B were, respectively, 2 days and 30 days after eclosion when imaged. Note that in F only anterograde motion of mitochondria was significantly reduced by RNAi of *Pka-C1*. Despite the specific decrease in anterograde transport, the total number of mitochondria in the axonal segment was not altered (Figure 2E). This finding is consistent with previous evidence that net transport is not the sole determinant of the steady-state distribution of mitochondria in axons [S1]. Statistical significance was evaluated with a Mann-Whitney U-test (A-E and G) and a one-way ANOVA with Dunnett's multiple comparisons correction (F). *P*-value: *, <0.05; **, <0.01; ****, <0.0001. Magenta circles are values for individual wings; error bars are SEM.

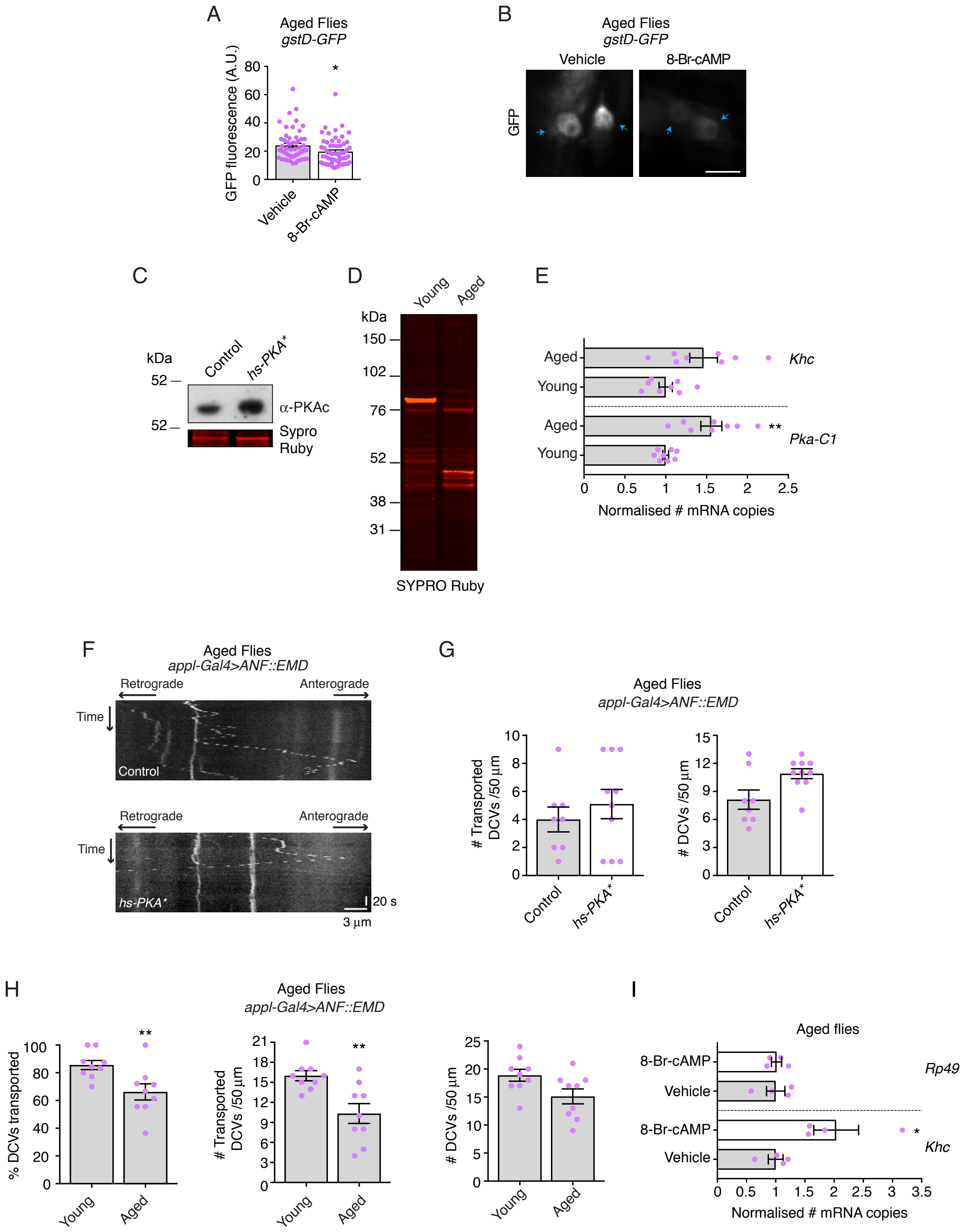

A
Aged Flies
gstD-GFP
GFP fluorescence (A.U.)
0
20
40
60
80
*
Vehicle
8-Br-cAMP
B
Aged Flies
gstD-GFP
Vehicle
8-Br-cAMP
GFP
C
kDa
52
52
Control
hs-PKA*
α-PKAc
Sypro Ruby
D
Young
Aged
kDa
150
102
76
52
38
31
SYPRO Ruby
E
Aged
Young
Aged
Young
Khc
Pka-C1
**
0
0.5
1
1.5
2
2.5
Normalised # mRNA copies
F
Aged Flies
appl-Gal4>ANF::EMD
Retrograde
Anterograde
Time
Control
Retrograde
Anterograde
Time
hs-PKA*
20 s
3 μm
G
Aged Flies
appl-Gal4>ANF::EMD
Transported DCVs /50 μm
0
2
4
6
8
10
Control
hs-PKA*
DCVs /50 μm
0
3
6
9
12
15
Control
hs-PKA*
H
Aged Flies
appl-Gal4>ANF::EMD
% DCVs transported
0
20
40
60
80
100
**
Young
Aged
Transported DCVs /50 μm
0
3
6
9
12
15
18
21
**
Young
Aged
DCVs /50 μm
0
5
10
15
20
25
Young
Aged
I
Aged flies
8-Br-cAMP
Vehicle
8-Br-cAMP
Vehicle
Rp49
Khc
*
0
0.5
1
1.5
2
2.5
3
3.5
Normalised # mRNA copies

Figure S2. Supplemental data on cAMP/PKA pathway activation. Related to Figures 1, 2 and 3.
(A) GFP fluorescence in cell bodies of wing neurons of 30-day-old *gstD-GFP* flies fed throughout adulthood with food supplemented with 8-Br-cAMP or standard food (vehicle). GFP is expressed in response to oxidative stress through activation of the *gstD* enhancer elements by the Nrf2 transcription factor [S2, S3].
(B) Images showing examples of GFP signals in cell bodies (blue arrows) of wing neurons expressing *gstD-GFP*. Scale bar: 5 µm.
(C) Top panel: Immunoblot for PKA* using extracts of heads of 4-day-old control (wild-type) or *hs-PKA** flies cultured at 30°C for 4 days. The more intense band in the *hs-PKA** sample confirms expression of PKA* from the transgene. Note that the antibody recognises both the endogenous *Drosophila* PKAc and exogenous mouse PKA*. Bottom panel shows a segment of SYPRO Ruby-stained gel from the same samples, which contains an abundant protein that is not responsive to PKA* induction.
(D) SYPRO Ruby-stained gel of wing extracts of young (day 2) and aged (day 30) wild-type flies.
(E) Abundance of *Pka-C1* and *Khc* mRNAs in young (2 day) and aged (day 30) wild-type flies. mRNA level was assessed by RT-ddPCR, with values normalised to those for the vehicle control. Magenta circles are values for individual technical replicates from two independent reverse transcription reactions. There was a statistically significant increase in *Pka-C1* mRNA in the aged sample compared to the young sample (1.5-fold difference), which could conceivably reflect a mechanism to compensate for reduced PKAc protein.
(F) Kymographs illustrating axonal transport of DCVs in wing neurons of heat-shocked control and *hs-PKA** aged flies.
(G) Quantification of the number of transported and total DCVs in the axonal tract in the wing of heat-shocked control and *hs-PKA** aged flies. There was a modest, but not statistically significant, increase in the total number of DCVs in axons when PKA* was overexpressed.
(H) Quantification of DCV transport in the axonal tract in the wing of young (day 2) and aged (day 30) animals. There was a statistically significant reduction in the percentage of DCVs that are transported in the aged flies compared to the young flies. We previously monitored DCV motility in wild-type wing neurons over the first 16 days of adult life and found no decline in transport [S4]; this contrasts with the sharp decline in mitochondrial transport observed during this period [S4].
In G and H, magenta circles are values for individual wings, with each wing filmed for 3 minutes.
(I) Abundance of *Khc* and *Rp49* mRNA in wings of aged wild-type flies (day 31) fed either vehicle or 8-Br-cAMP for the preceding 24 hours. mRNA abundance was assessed by RT-ddPCR, with values normalised to those for the vehicle control.
Statistical significance was evaluated with the Mann-Whitney U-test. *P*-values: *, <0.05; **, <0.01; error bars are SEM.

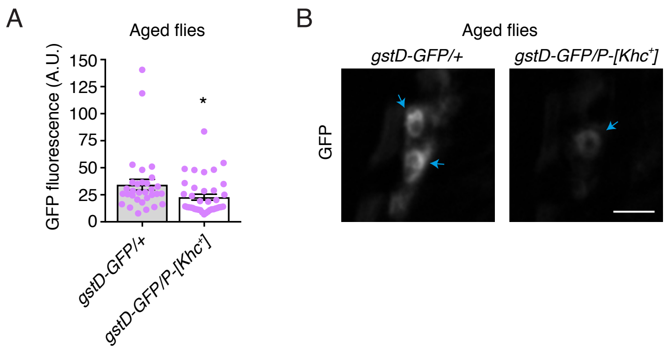

Figure S3. Supplemental data on Khc overexpression. Related to Figure 4.
(A) Quantification of GFP fluorescence in cell bodies of wing neurons of 30-day-old *gstD-GFP/+* or *gstD-GFP/P[Khc$^+$]* flies. Magenta circles are values for individual Z-projections from a total of 10 wings of each genotype. Statistical significance was evaluated with a two-tailed Student's *t*-test. *P*-values: *, <0.05; error bars are SEM.
(B) Images showing examples of GFP signals in cell bodies (arrows) of wing neurons expressing *gstD-GFP*. Scale bar: 5 µm.

	Mean velocity (µm s^{-1})		Mean run length (µm)	
Condition	Anterograde	Retrograde	Anterograde	Retrograde
**Related to Fig. 1**				
Aged flies:				
luc^{RNAi}	0.19 ± 0.04 (9)	NA (0)	7.49 ± 1.93 (9)	NA (0)
dnc^{RNAi}	0.17 ± 0.02 (20)	0.33 ± 0.04 (15)	12.00 ± 1.71 (20)	10.80 ± 1.48 (15)
Young flies:				
Vehicle	0.20 ± 0.01 (37)	0.32 ± 0.03 (24)	14.21 ± 1.74 (37)	11.00 ± 1.57 (24)
8-Br-cAMP	0.20 ± 0.01 (47)	0.33 ± 0.04 (17)	10.97 ± 1.17 (47)	11.65 ± 2.71 (17)
Aged flies:				
Vehicle	0.23 ± 0.02 (15)	0.47 ± 0.05 (7)	16.96 ± 2.99 (15)	7.99 ± 2.64 (7)
8-Br-cAMP	0.24 ± 0.01 (31)	0.35 ± 0.06 (16)	15.79 ± 1.51 (31)	10.21 ± 1.56 (16)
**Related to Fig. 2**				
Young flies:				
Control	0.19 ± 0.01 (40)	0.33 ± 0.05 (20)	16.66 ± 1.3 (40)	10.93 ± 1.56 (20)
*hs-PKA**	0.25 ± 0.01 (52) **	0.31 ± 0.04 (19)	16.90 ± 1.35 (52)	14.90 ± 2.95 (19)
Aged flies:				
Control	0.22 ± 0.02 (24)	0.29 ± 0.06 (9)	12.14 ± 1.95 (24)	11.88 ± 2.99 (9)
*hs-PKA**	0.23 ± 0.02 (49)	0.38 ± 0.05 (18)	10.91 ± 1.02 (49)	13.45 ± 2.07 (18)
Young flies:				
luc^{RNAi}	0.21 ± 0.01 (78)	0.39 ± 0.05 (17)	12.25 ± 1.01 (78)	12.19 ± 2.55 (17)
Pka-C1^{RNAi1}	0.28 ± 0.02 (37) ***	0.37 ± 0.06 (12)	12.71 ± 1.31 (37)	13.39 ± 2.47 (12)
Pka-C1^{RNAi2}	0.29 ± 0.01 (40) ***	0.55 ± 0.07 (16)	12.18 ± 1.10 (40)	8.60 ± 1.21 (16)
**Related to Fig. 4**				
Young flies:				
Control	0.18 ± 0.01 (104)	0.33 ± 0.03 (45)	12.96 ± 0.84 (104)	11.39 ± 0.87 (45)
P[Khc^{+}*]*	0.21 ± 0.07 (110) *	0.37 ± 0.03 (59)	12.41 ± 0.87 (110)	13.81 ± 1.31 (59)
Aged flies:				
Control	0.20 ± 0.02 (19)	0.23 ± 0.06 (6)	13.56 ± 2.00 (19)	8.12 ± 2.42 (6)
P[Khc^{+}*]*	0.19 ± 0.01 (36)	0.35 ± 0.05 (16)	12.45 ± 1.39 (36)	9.07 ± 1.91 (16)

Table S1. **Velocity and run length of motile mitochondria after manipulation of the cAMP/PKA pathway or Khc overexpression. Related to Figures 1, 2 and 4.**

Number of runs are in parentheses.

NA: not applicable as no retrograde movements were observed.

For comparisons involving two groups, statistical significance was evaluated with a Mann-Whitney U test (when $n < 10$) or two-tailed Student's t-test (when $n > 10$). An ANOVA test with Dunnett's multiple comparison correction was used for *Pka-C1* RNAi experiments, which involved three groups. *P*-value: *, <0.05; **, <0.01; ***, <0.0001; errors are SEM.

Both RNAi treatment and overexpression of PKAc significantly increased anterograde velocity in young flies, suggesting complex regulation of this parameter at this stage.

Mean run lengths were not significantly affected by changes in Khc concentration, consistent with travel distances of certain cargoes being insensitive to changes in the number of associated kinesin-1 motors [S5, S6].

Name	Sequence
Khc (FWD)	CAGAACATCATCCTCACCAAC
Khc (RVS)	CCTCGCTCTCCTCGTTTAC
Rp49 (FWD)	CGGAAACTCAATGGATACTGC
Rp49 (RVS)	CGACTGGTGGCGGATGAAGTG
Rap2L (FWD)	ACTTCCGTGCATTACGTGCG
Rap2L (RVS)	CCGACCCGAGCACAACAACT
14-3-3 (FWD)	CATGAACGATCTGCCACCAAC
14-3-3 (RVS)	CTCTTCGCTCAGTGTATCCAAC
eIF-1A (FWD)	ATCAGCTCCGAGGATGACGC
eIF-1A (RVS)	GCCGAGACAGACGTTCCAGA
Milton (FWD)	CAGGATCAGCTGAAGCAACA
Milton (RVS)	AGCTCACAGTGCAGTGAATCC
Miro (FWD)	ACTCATCTAATCCCCGTTCC
Miro (RVS)	GCAGCAACTTGTACTTGTCAC
Pka-C1 (FWD)	GGATCAGCCGATTCAGATCTAC
Pka-C1 (RVS)	AATATCATTGACGCCCGCC
Sgg (FWD)	CTGGTAACAACAGTAGCAGC
Sgg (RVS)	CATTAGTCGCGCCTATAGC

Table S2. **Details of gene-specific primers used for RT-ddPCR. Related to Figures 3, S2 and Key Resources Table.**

Supplemental References

S1. Lopez-Domenech, G., Higgs, N.F., Vaccaro, V., Ros, H., Arancibia-Carcamo, I.L., MacAskill, A.F., and Kittler, J.T. (2016). Loss of dendritic complexity precedes neurodegeneration in a mouse model with disrupted mitochondrial distribution in mature dendrites. Cell Rep. 17, 317-327.

S2. Sykiotis, G.P., and Bohmann, D. (2008). Keap1/Nrf2 signaling regulates oxidative stress tolerance and lifespan in *Drosophila*. Dev. Cell 14, 76-85.

S3. Milton, V.J., Jarrett, H.E., Gowers, K., Chalak, S., Briggs, L., Robinson, I.M., and Sweeney, S.T. (2011). Oxidative stress induces overgrowth of the *Drosophila* neuromuscular junction. Proc. Natl. Acad. Sci. USA 108, 17521-17526.

S4. Vagnoni, A., Hoffmann, P.C., and Bullock, S.L. (2016). Reducing Lissencephaly-1 levels augments mitochondrial transport and has a protective effect in adult *Drosophila* neurons. J. Cell Sci. 129, 178-190.

S5. Shubeita, G.T., Tran, S.L., Xu, J., Vershinin, M., Cermelli, S., Cotton, S.L., Welte, M.A., and Gross, S.P. (2008). Consequences of motor copy number on the intracellular transport of Kinesin-1-driven lipid droplets. Cell 135, 1098-1107.

S6. Jamison, D.K., Driver, J.W., Rogers, A.R., Constantinou, P.E., and Diehl, M.R. (2010). Two kinesins transport cargo primarily via the action of one motor: implications for intracellular transport. Biophys. J. 99, 2967-2977.
