# Supplementary material for: A cAMP/PKA/Kinesin-1 Axis Promotes the Axonal Transport of Mitochondria in Aging Drosophila Neurons
Source: Curr Biol. 2018 Apr 23;28(8):1265–1272.e4. doi: 10.1016/j.cub.2018.02.048 (PMC5912900; doi:10.1016/j.cub.2018.02.048)
Supplement: Document S2. Article plus Supplemental Information [file mmc6.pdf]

Report

Current Biology

A cAMP/PKA/Kinesin-1 Axis Promotes the Axonal Transport of Mitochondria in Aging *Drosophila* Neurons

Graphical Abstract

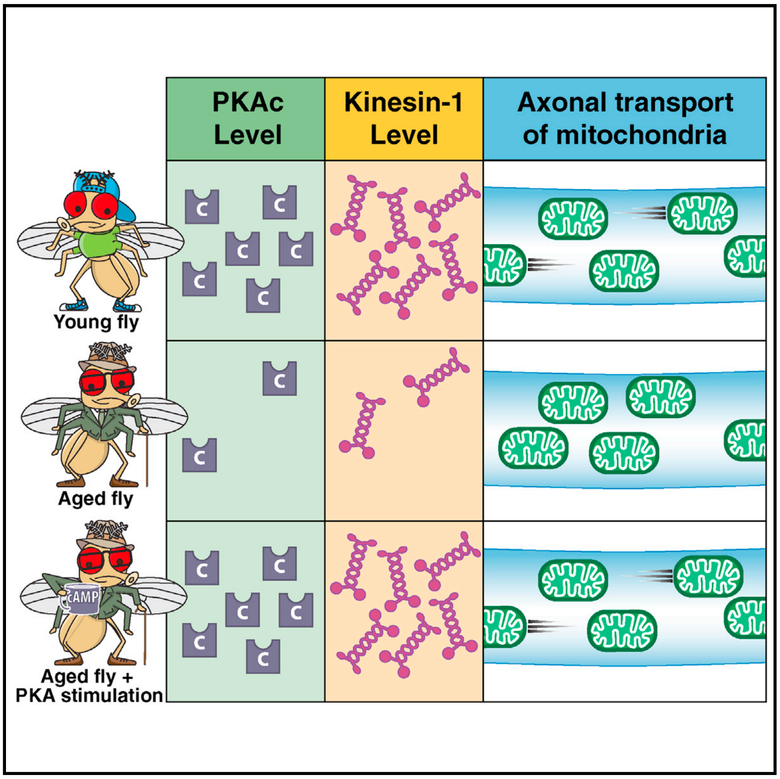

Highlights

- cAMP/PKA pathway promotes mitochondrial transport in adult *Drosophila* wing neurons
- Pathway activation in aged flies suppresses age-related reduction in transport
- Levels of PKAc and kinesin-1 motor decline during aging
- Kinesin-1 upregulation is an important output of PKA activation in aged flies

Authors

Alessio Vagnoni, Simon L. Bullock

Correspondence

alessio.vagnoni@kcl.ac.uk (A.V.),
sbullock@mrc-lmb.cam.ac.uk (S.L.B.)

In Brief

Vagnoni and Bullock use the *Drosophila* wing to investigate the regulation of mitochondrial transport in aging neurons. They discover that acute activation of the cAMP/PKA pathway can suppress an age-related decline in mitochondrial motility. This effect is due in part to upregulation of the kinesin-1 motor, levels of which decline during aging.

Vagnoni & Bullock, 2018, Current Biology *28*, 1265–1272
April 23, 2018 © 2018 The Author(s). Published by Elsevier Ltd.
https://doi.org/10.1016/j.cub.2018.02.048

Current Biology
Report

CellPress

A cAMP/PKA/Kinesin-1 Axis Promotes the Axonal Transport of Mitochondria in Aging *Drosophila* Neurons

Alessio Vagnoni[1,2,3,*] and Simon L. Bullock[1,*]
[1]Division of Cell Biology, MRC Laboratory of Molecular Biology, Cambridge CB2 0QH, UK
[2]Department of Basic and Clinical Neuroscience, Maurice Wohl Clinical Neuroscience Institute, King's College London, London SE5 9RX, UK
[3]Lead Contact
*Correspondence: alessio.vagnoni@kcl.ac.uk (A.V.), sbullock@mrc-lmb.cam.ac.uk (S.L.B.)
https://doi.org/10.1016/j.cub.2018.02.048

SUMMARY

Mitochondria play fundamental roles within cells, including energy provision, calcium homeostasis, and the regulation of apoptosis. The transport of mitochondria by microtubule-based motors is critical for neuronal structure and function. This process allows local requirements for mitochondrial functions to be met and also facilitates recycling of these organelles [1, 2]. An age-related reduction in mitochondrial transport has been observed in neurons of mammalian and non-mammalian organisms [3–6], and has been proposed to contribute to the broader decline in neuronal function that occurs during aging [3, 5–7]. However, the factors that influence mitochondrial transport in aging neurons are poorly understood. Here we provide evidence using the tractable *Drosophila* wing nerve system that the cyclic AMP/protein kinase A (cAMP/PKA) pathway promotes the axonal transport of mitochondria in adult neurons. The level of the catalytic subunit of PKA decreases during aging, and acute activation of the cAMP/PKA pathway in aged flies strongly stimulates mitochondrial motility. Thus, the age-related impairment of transport is reversible. The expression of many genes is increased by PKA activation in aged flies. However, our results indicate that elevated mitochondrial transport is due in part to upregulation of the heavy chain of the kinesin-1 motor, the level of which declines during aging. Our study identifies evolutionarily conserved factors that can strongly influence mitochondrial motility in aging neurons.

RESULTS AND DISCUSSION

To shed light on the regulation of mitochondrial transport in aging neurons, we exploited a tractable system for imaging of axonal transport in an adult animal: the sensory neurons of the translucent *Drosophila* wing [5, 8, 9] (Figure 1A). We previously showed that the number of actively transported mitochondria in wing neuron axons is ∼5-fold lower at 30 days after eclosion from the pupal case (operationally defined as "aged" flies) than at 2 days after eclosion ("young" flies) [5] (Figure S1A). The decline in mitochondrial transport begins in the first week of adult life [5] and reflects reduced anterograde and retrograde movements (Figure S1B), equating to transport toward microtubule plus ends and minus ends, respectively [5]. The number of mitochondria in axonal tracts does not change during aging, demonstrating that a larger fraction of mitochondria becomes stationary over time [5].

Signaling pathways that increase lifespan are attractive candidates for exploring the regulation of axonal transport during aging [6, 10]. Cyclic AMP (cAMP) is an important second messenger in intracellular signaling, and elevation of its concentration can extend lifespan in *Drosophila* and mice [11, 12]. We therefore investigated whether cAMP influences axonal transport of mitochondria in aged flies.

We first targeted the cAMP phosphodiesterase Dunce (Dnc) in wing neurons using an RNA interference (RNAi) construct under the control of the Gal4-UAS system. Downregulation of Dnc increases the concentration of cAMP by inhibiting conversion to AMP [13]. To visualize mitochondria, we co-expressed GFP targeted to the mitochondrial matrix (mito::GFP). In aged flies, the number of transported mitochondria in axons of *dnc* RNAi neurons was ∼4-fold higher than in neurons expressing a control RNAi construct (Figures 1B and 1C; Movie S1). RNAi of *dnc* increased transport in both the anterograde and retrograde directions (Figure S1C) without altering the number of mitochondria in the axonal tract (Figure 1C). Thus, prolonged inhibition of cAMP turnover in wing neurons of aged flies increases mitochondrial transport by mobilizing a fraction of stationary mitochondria.

Aging is characterized by a functional decline at the cellular and organismal level. A key question in aging research is to what extent prolonged reduction of cellular functions can be rescued by an acute intervention in later life [14]. We therefore asked whether an acute increase in cAMP can boost mitochondrial transport in aged flies. 28-day-old flies were fed with 100 μM 8-Br-cAMP (a hydrolysis-resistant cAMP analog) for 4 days prior to visualization of mitochondrial motility in wing neuron axons (Figure 1D). The number of motile mitochondria was significantly higher in flies fed with 8-Br-cAMP than in vehicle-fed controls (Figures 1E and 1F; Movie S2). Again, the increase in motility was evident in both the anterograde and retrograde directions (Figure S1D), with no change in the number of

This is an open access article under the CC BY license (http://creativecommons.org/licenses/by/4.0/).

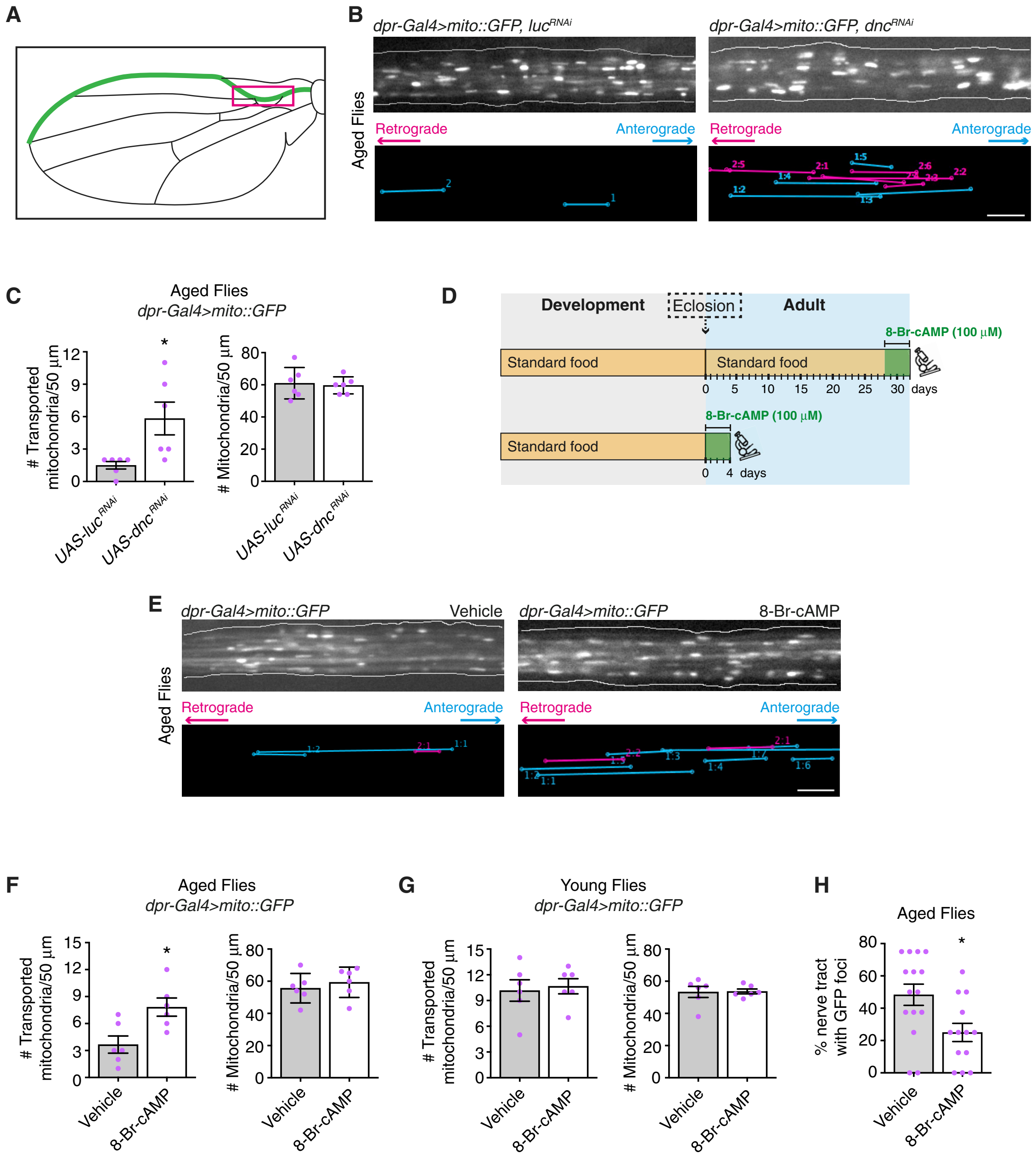

Figure 1. cAMP Boosts Mitochondrial Transport in Wing Neurons of Aged Flies

(A) Cartoon of the *Drosophila* wing. Green: sensory neurons used in this study. Magenta box: region imaged for transport studies, which contains bundled axons from several neurons.

(B) Top: stills from movies of GFP-labeled mitochondria in wing neuron axons of 30-day-old flies ("aged" flies) expressing *UAS-luciferase (luc)*^{RNAi} (control) or *UAS-dnc*^{RNAi}. Bottom: traces of transported mitochondria in corresponding movies.

(C) Number of transported and total mitochondria per 50 μm of axonal tract in wings of aged flies. In these and other experiments, each wing was filmed for 3 min.

(D) Overview of 8-Br-cAMP feeding experiments. Green square: period of feeding. Microscope icon: visualization of mitochondrial transport.

(E) Top: stills from movies of GFP-labeled mitochondria in wing neuron axons of flies 32 days after eclosion following 4 days of feeding with vehicle or 8-Br-cAMP. Bottom: traces of transported mitochondria in corresponding movies.

(F and G) Number of transported and total mitochondria per 50 μm of axonal tract of wing neurons in aged (F) and young (G) flies after 4 days of feeding with vehicle or 8-Br-cAMP.

(H) Percentage of axonal segments of wing neurons in 5-week-old flies that contained focal accumulation of GFP after feeding throughout adulthood with vehicle or 8-Br-cAMP.

dpr-Gal4 is expressed in the chemosensory neurons of the wing. Statistical significance was evaluated with a Mann-Whitney U test (C, F, and G) or two-tailed Student's t test (H). *$p < 0.05$. Magenta circles are values for individual wings, except in (H), where they are values from individual Z projections from 13 wings per genotype; error bars are SEM. Scale bars, 5 μm. See also Figures S1 and S2, Movies S1 and S2, and Table S1.

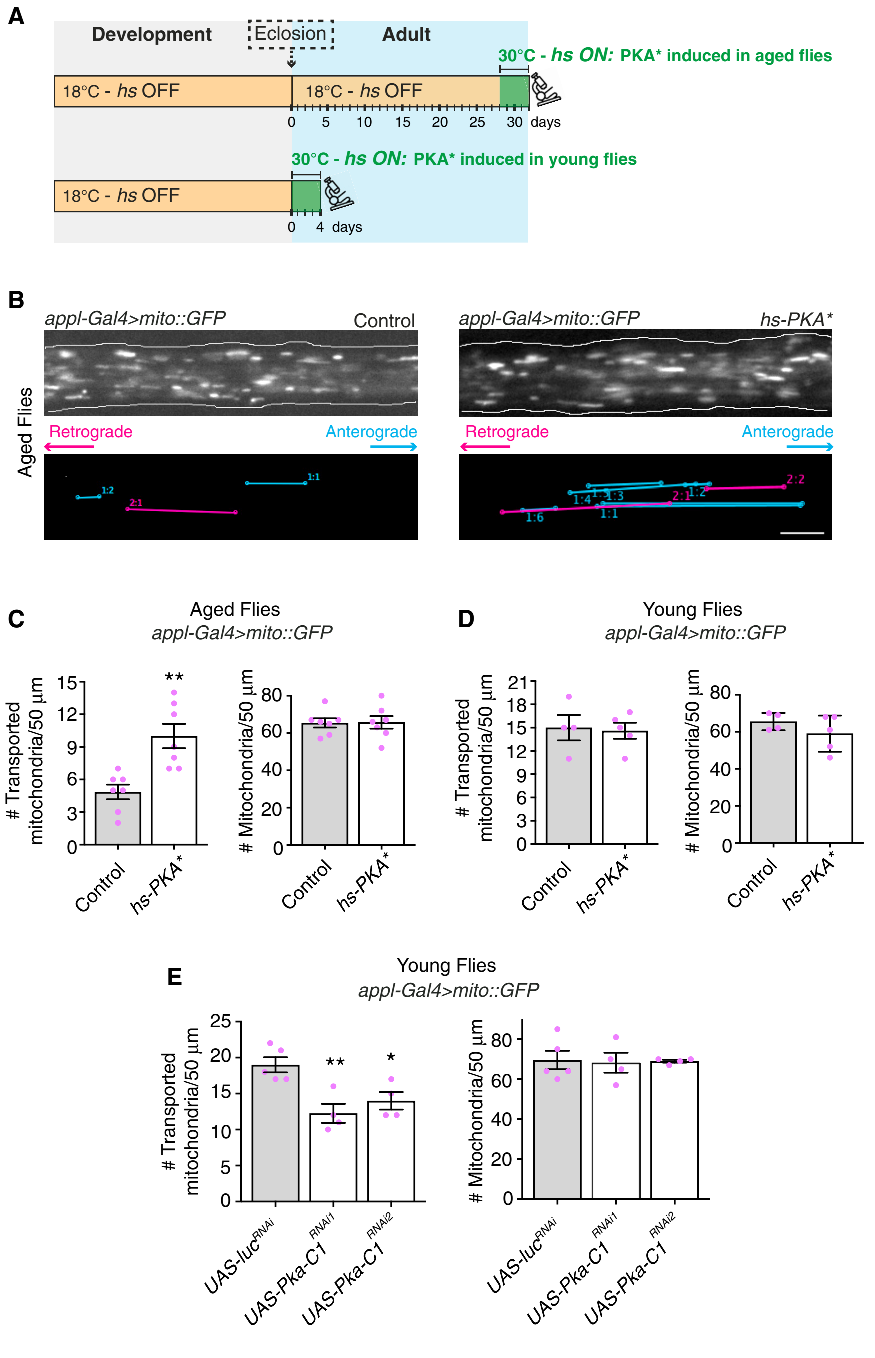

Figure 2. PKA-Induced Upregulation of Mitochondrial Transport in Wing Neurons of Aged Flies

(A) Overview of the procedure for heat-shock (hs)-mediated induction of PKA*. Green square: period of heat shock. Microscope icon: visualization of mitochondrial transport.

(B) Top: stills from movies of GFP-labeled mitochondria in wing neuron axons of aged control and *hs-PKA** flies that have been subjected to heat shock. Bottom: traces of transported mitochondria in corresponding movies.

(C and D) Number of transported and total mitochondria per 50 μm of axonal tract of wing neurons in aged (C) and young (D) control or *hs-PKA** flies subjected to heat shock.

(E) Number of transported and total mitochondria per 50 μm of axonal tract of wing neurons of 2-day-old flies with expression of *UAS-luciferase^{RNAi}* or two independent *UAS-Pka-C1^{RNAi}* constructs.

appl-Gal4 marks both the mechanosensory and chemosensory neurons in the wing nerve. Statistical significance was evaluated with a Mann-Whitney U test (C and D) or a one-way ANOVA with Dunnett's multiple comparison (E). *$p < 0.05$, **$p < 0.01$. Magenta circles are values for individual wings; error bars are SEM. Scale bar, 5 μm. See also Figures S1 and S2, Movie S3, and Table S1.

mitochondria in the axonal tract (Figures 1F). The number of transported mitochondria in wing neurons of aged flies fed with 8-Br-cAMP was not significantly different from that observed in flies fed with the vehicle control for the first 4 days of adulthood (Figures 1F and 1G). Interestingly, feeding flies with 8-Br-cAMP for the first 4 days of adult life (Figure 1D) did not alter the number of motile mitochondria compared to the age-matched controls fed with vehicle (Figure 1G). These results demonstrate that acute supply of cAMP can increase mitochondrial motility in aged but not young flies.

We next investigated whether elevating cAMP levels has broader effects on wing neurons of aged flies. Sustained feeding with 8-Br-cAMP reduced the appearance of focal accumulations of GFP (Figure 1H), indicative of improved protein homeostasis [5]. 8-Br-cAMP treatment also reduced the signal from an oxidative stress sensor (Figures S2A and S2B). Although the cAMP analog is likely to affect many processes in wing neurons of aged flies, these results are consistent with previous evidence that mitochondrial transport has a protective role in adult neurons of *Drosophila* [5, 8, 15], *C. elegans* [6, 16], and mice [17].

Several studies have reported that cAMP concentration modulates mitochondrial transport in cultured mammalian cells [18–20], although the underlying mechanisms have not been resolved. A key cellular role of cAMP is activation of the catalytic subunit of protein kinase A (PKAc) by triggering dissociation of the regulatory subunit. We investigated whether cAMP-mediated stimulation of mitochondrial transport in wing neurons involves PKA by expressing a constitutively active mouse PKAc (PKA*) from a heat-shock-responsive promoter (*hs-PKA**) (Figure 2A; Figure S2C). The induction of PKA* in aged flies significantly increased the number of transported mitochondria compared to controls that lacked the transgene but were subjected to the same heat-shock regime (Figures 2B and 2C; Movie S3). This effect was associated with more frequent retrograde and anterograde movements (Figure S1E) and no change in the number of mitochondria in the axonal tract (Figures 2B and 2C). In contrast, expressing PKA* in

young flies (Figure 2A) did not alter the number of transported mitochondria (Figure 2D). The outcomes of PKA* expression in young and aged flies were very similar to those observed after feeding with 8-Br-cAMP, indicating that cAMP-mediated stimulation of mitochondrial transport in aged flies involves PKA.

We assessed the role of endogenous PKA during mitochondrial transport in wing neurons by targeting *Pka-C1* mRNA, which encodes the major catalytic PKA subunit in *Drosophila* (PKAc). Two different *Pka-C1* RNAi constructs reduced the number of transported mitochondria in young flies without altering mitochondrial number in the axonal tract (Figure 2E; Figure S1F). These experiments demonstrate that endogenous PKA activity promotes axonal transport of mitochondria. However, levels of PKA activation must not be limiting in young flies, because mitochondrial transport was insensitive to experimental elevation of cAMP or PKA*.

We next asked whether cAMP/PKA pathway activity could become limiting for transport in aged flies because of declining levels of cAMP or PKAc. Adult wings were used as a source of cell extracts for these experiments, because wing neurons constitute a large fraction of the living material in this sclerotized tissue [5, 21]. Whereas the concentration of cAMP in the wings of aged flies did not decline compared to those of young flies (Figure 3A), the level of PKAc did (Figure 3B). The reduction in PKAc level was not associated with a general decrease in protein abundance in aged flies (Figure 3B; Figure S2D). The level of *Pka-C1* mRNA in wings was not lower in aged flies compared to young flies (Figure S2E), indicating that the decline in PKAc abundance is due to altered synthesis or turnover of the protein. Although we cannot rule out other mechanisms, a reduction in PKAc protein levels could explain why cAMP/PKA pathway activity is limiting for mitochondrial transport in aged flies. Presumably, increasing cAMP levels compensate for the reduction in PKAc concentration in aged neurons by activating the protein that is available.

We then explored how experimental elevation of cAMP/PKA upregulates mitochondrial transport in aging wing neurons. To test whether increased movement of mitochondria reflects a general increase in cargo transport, we monitored the transport of fluorescently labeled dense-core vesicles (DCVs) after heat-shock-mediated induction of PKA*. In contrast to what was observed for mitochondria, expression of PKA* in aged flies did not increase the frequency of DCV transport compared to heat-shocked controls (Figure 3C; Figures S2F and S2G). Although effects on motility of other cargoes remain to be tested, this experiment demonstrates that not all microtubule-based transport is elevated by PKA* expression. The selective effect of PKA* overexpression on transport of mitochondria and DCVs is consistent with these cargoes being differentially affected during aging, with DCVs only exhibiting a small reduction in motility during the first 30 days of adult life [5] (Figure S2H).

Although mitochondria and DCVs both use dynein for retrograde axonal transport in *Drosophila* neurons [5, 24, 25], they differ in the identity of the motors primarily used for anterograde motion. Anterograde movement of mitochondria is driven by kinesin-1 [5, 24], whereas DCVs use kinesin-3/Unc104 [25]. We therefore asked whether the sensitivity of mitochondria to normal aging and the experimental elevation of PKA activity could be related to their reliance on kinesin-1.

We observed a reduction in the level of the Kinesin-1 motor subunit (Kinesin-1 heavy chain, Khc) in wings of aged flies compared to wings of young flies (Figure 3D). This reduction must be due to altered synthesis or turnover of the protein, as *Khc* mRNA abundance did not decline with age (Figure S2E). These findings raised the possibility that reduced Khc abundance contributes to the decline in mitochondrial transport in aged flies. Limiting levels of Khc could in principle account for the age-related reduction in retrograde and anterograde motion because the activity of dynein in this system depends on kinesin-1 [5], as is the case elsewhere [26].

Next, we asked whether the level of Khc is altered by experimental activation of the cAMP/PKA pathway in aged flies. We observed a strong increase in Khc protein abundance in wing extracts of aged animals following heat-shock-mediated induction of PKA* (Figure 3E). Gel-based analysis of protein extracts revealed that several abundant proteins were also upregulated by PKA* expression, although the levels of other abundant proteins did not increase (Figure 3F). Thus, Khc is one of a number of proteins upregulated by cAMP/PKA pathway activation in aged flies.

cAMP/PKA pathway activation increases levels of *Khc* mRNA in *Aplysia* neurons [27]. We therefore monitored *Khc* mRNA abundance in wings of aged flies with heat-shock-mediated induction of PKA* or with heat-shock treatment alone. There was an ~6.5-fold increase in the amount of *Khc* transcript in response to PKA* expression, whereas the level of the mRNA encoding a ‘‘housekeeping’’ protein (ribosomal protein 49, Rp49) did not change (Figure 3G). *Khc* mRNA, but not *Rp49* mRNA, was also upregulated by acute supply of 8-Br-cAMP to aged flies (Figure S2I). Thus, unlike the decline in Khc protein levels during aging, the increase in Khc protein levels upon experimental activation of cAMP/PKA is associated with changes in mRNA abundance. The best-characterized mechanism for PKA-mediated gene expression involves the cAMP response element-binding protein (CREB) transcription factor. CREB activity is stimulated by phosphorylation, which can be mediated by multiple kinases, including PKA [28]. The abundance of *Khc* mRNA in aged flies was decreased by heat-shock-mediated induction of a dominant-negative CREB transgene (*hs-dCREB2*-b; Figure 3H). By providing evidence that the *Khc* gene is responsive to CREB, these data suggest a mechanism for upregulation of *Khc* mRNA upon experimental activation of PKA in aged flies.

Khc is linked to mitochondria by the concerted action of two proteins, Milton and Miro [1]. The level of *Milton* mRNA in aged wings was not affected by PKA*, whereas the level of *Miro* RNA was increased by 1.7-fold (Figure 3G). Thus, of the mRNAs encoding components of the anterograde mitochondrial transport complex, the one encoding *Khc* is most responsive to cAMP/PKA pathway activation. The level of *Pka-C1* mRNA was also upregulated by PKA* (Figure 3G), consistent with the ability of PKAc to stimulate its own expression in mammalian cells [29]. Several other mRNAs tested also increased in abundance in response to PKA*, although the response was weaker than observed for *Khc* mRNA (Figure 3G). These results are consistent with the widespread transcriptional changes induced by PKA in mammalian cells [30, 31].

Our finding that many mRNAs are upregulated by PKA activation in aging wings raised the question of whether the increased abundance of Khc plays a significant role in boosting

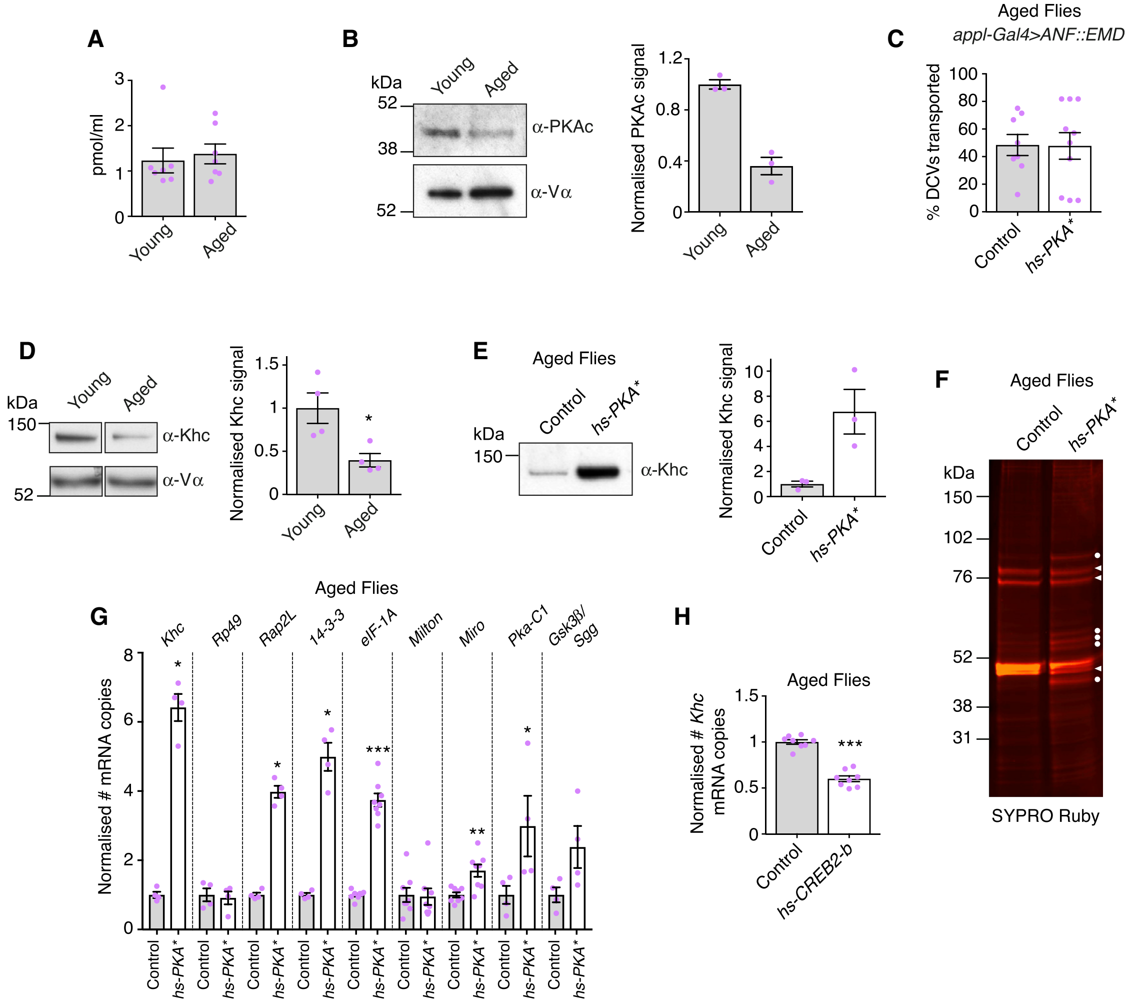

Figure 3. Overexpression of Activated PKAc in Aged Flies Upregulates Khc Protein and mRNA

(A) cAMP levels in wings of young (day 2) and aged (day 30) wild-type flies assessed by ELISA. Magenta circles are values per milliliter of extract (technical replicates from two independent experiments).

(B) Representative immunoblot for PKAc and mitochondrial complex-Vα (Vα) using wing extracts of young (day 2) and aged (day 30) wild-type flies. Vα signal indicates no global decline in protein levels during aging. Charts show quantification of normalized PKAc signal from three independent experiments.

(C) Percentage of DCVs that are transported in the axonal tract of wing neurons in heat-shocked control or *hs-PKA** flies. Magenta circles are values for individual wings. DCVs were marked with rat prepro-atrial natriuretic factor peptide fused to the fluorescent protein Emerald (ANF::EMD).

(D) Representative immunoblot for Khc and Vα using wing extracts of young (day 2) and aged (day 30) wild-type flies. Chart shows quantification of normalized Khc signal from four independent experiments.

(E) Representative immunoblots for Khc using wing extracts of 32-day-old control or *hs-PKA** flies following 4-day heat shock. Chart shows quantification of normalized Khc signal from three independent experiments.

(F) SYPRO Ruby-stained gel of wing extracts of 32-day-old heat-shocked control or *hs-PKA** used in (E). Circles and arrowheads indicate, respectively, abundant proteins that are or are not responsive to PKA*.

(G) Reverse-transcription digital droplet-PCR (RT-ddPCR) analysis of the relative abundance of mRNAs in wings of control and *hs-PKA** 31-day-old flies subjected to heat shock for the preceding 24 hr. Magenta circles are values from individual technical replicates from two independent reverse-transcription reactions per genotype. Rp49 has been used as a “housekeeping” gene in RT-PCR experiments with wings [22]. *Rap2L*, *14-3-3*, and *eIF-1A* mRNAs were previously detected in RT-PCR experiments in *Drosophila* heads [23]. Sgg/Gsk3β upregulates anterograde mitochondrial motility in mammalian neurons [20].

(H) RT-ddPCR analysis of the relative abundance of *Khc* mRNA in wings of 32-day-old control and *hs-dCREB2-b* flies following 4 days of heat shock. Magenta circles are values from individual technical replicates.

Statistical significance was evaluated with the Mann-Whitney U test. *p < 0.05, **p < 0.01, ***p < 0.001. Error bars are SEM. See also Figure S2 and Table S2.

mitochondrial transport. We therefore asked whether Khc overexpression is sufficient to boost mitochondrial motility in wing neurons of aged flies. For these experiments, we used a *Drosophila Khc* genomic rescue construct (*P[Khc$^+$]*) to increase Khc protein levels (Figure 4A).

P[Khc$^+$] increased the number of mitochondrial movements in aged flies by 2.1-fold without altering the number of mitochondria in the axonal tract (Figures 4B and 4C; Movie S4). The boost in transport was associated with an increase in both retrograde and anterograde movements (Figure S1G), again consistent

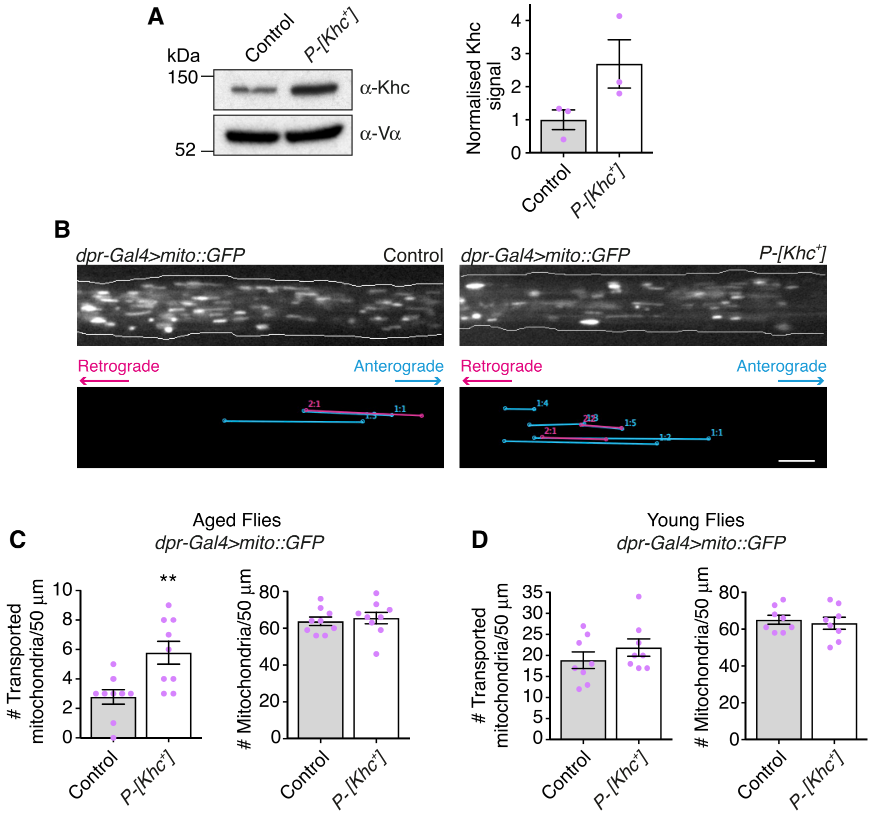

Figure 4. Khc Overexpression Is Sufficient to Increase Mitochondrial Transport in Aged Flies

(A) Representative immunoblots for Khc and Vα using wing extracts of 30-day-old wild-type or *P[Khc⁺]* flies. Chart shows quantification of normalized Khc signal from three independent experiments.

(B) Top: stills from movies of GFP-labeled mitochondria in wing neuron axons of aged (day 30) control and *P[Khc⁺]* flies. Bottom: traces of transported mitochondria in corresponding movies. Scale bar, 5 µm.

(C and D) Number of transported and total mitochondria per 50 µm of axonal tract of wing neurons in 30-day-old (C) and 2-day-old (D) control and *P[Khc⁺]* flies.

Statistical significance was evaluated with the Mann-Whitney U test. $^{**}p < 0.01$. Magenta squares: values for individual wings. Error bars are SEM. See also Figures S1 and S3, Movie S4, and Table S1.

with the tightly coupled activities of dynein and kinesin-1 in this system. Khc overexpression did not strongly affect the length and velocity of mitochondrial movements, as was also the case following manipulation of the cAMP/PKA pathway (Table S1). Thus, both types of intervention predominantly affect the initiation of transport. *P[Khc⁺]* also reduced the signal from an oxidative stress sensor in wing neurons of aged flies (Figure S3). This finding is consistent with protective effects of mitochondrial transport in this system [5], although elevated transport of other kinesin-1 cargoes could also contribute to this phenotype. It will be important in the future to understand the specific contribution of upregulated mitochondrial motility to energy metabolism in the wing nerve.

The frequency of mitochondrial transport in wing neurons of young flies was not increased by *P[Khc⁺]* (Figure 4D). The finding that kinesin-1 activity was not limiting at this stage is consistent with the endogenous level of Khc protein being higher than in aged flies (Figure 3D). Although we cannot rule out additional influences of activated PKA, such as direct phosphorylation of proteins that affect mitochondrial dynamics [32–37], our data indicate that Khc upregulation is a key functional output of experimental activation of PKA in the context of mitochondrial transport in aged neurons.

Perspective

Reduced axonal transport of mitochondria has been implicated in the pathogenesis of several age-related neurodegenerative diseases [38, 39]. It has therefore been proposed that stimulating mitochondrial transport could be of therapeutic benefit, potentially in combination with agents that target other cellular processes [38–41]. However, it was not clear to what extent a chronic decline in mitochondrial motility can be reversed. We find that feeding aged *Drosophila* with a small-molecule agonist of PKA is sufficient to ameliorate a decline in mitochondrial transport that begins in the first few days of adult life.

We provide evidence that the stimulation of transport upon cAMP/PKA pathway activation is associated with upregulation of the kinesin-1 motor subunit, which counteracts the reduction in levels of this protein that occurs during aging. The observation that cytosolic levels of the Khc ortholog decline in brains of aging monkeys [42] suggests that the regulatory processes we have uncovered in *Drosophila* could be of relevance in mammalian systems. Recent studies in mammalian neurons have shown that a progressive decrease in mitochondrial motility promotes developmental maturation of neurons [43–45]. It is tempting to speculate that whereas this attenuation of mitochondrial transport plays a positive role in early life, it becomes detrimental during aging when the functional demands of neurons change or stresses increase.

STAR★METHODS

Detailed methods are provided in the online version of this paper and include the following:

- KEY RESOURCES TABLE
- CONTACT FOR REAGENT AND RESOURCE SHARING
- EXPERIMENTAL MODEL AND SUBJECT DETAILS
  - *Drosophila* strains and husbandry
- METHOD DETAILS
  - Axonal transport assays
  - 8-Br-cAMP feeding

- ○ Immunoblotting and protein staining
- ○ Assessment of cAMP levels
- ○ RT-ddPCR
- ○ Oxidative stress and focal protein accumulation assays
- ● QUANTIFICATION AND STATISTICAL ANALYSIS

SUPPLEMENTAL INFORMATION

Supplemental Information includes three figures, two tables, and four movies and can be found with this article online at https://doi.org/10.1016/j.cub.2018.02.048.

ACKNOWLEDGMENTS

We thank Patrick Hoffmann for initial *dnc* RNAi experiments, Stefano Giandomenico for assistance with reverse-transcription digital droplet-PCR (RT-ddPCR), members of the *Drosophila* community for sharing reagents, the Wohl Cellular Imaging Centre at King's College London for help with light microscopy, MRC-LMB Visual Aids for artwork, and Giampietro Schiavo for support and critically reading the manuscript. This work was funded by the MRC (file reference number MC_U105178790; to S.L.B.), an NC3Rs David Sainsbury Fellowship (NC/N001753/1), and a Van Geest Fellowship in Dementia and Neurodegeneration (both to A.V.).

AUTHOR CONTRIBUTIONS

A.V. performed experiments and analyzed data; A.V. and S.L.B. conceived the study, designed experiments, and wrote the paper; and S.L.B. supervised the study.

DECLARATION OF INTERESTS

The authors declare no competing interests.

Received: September 5, 2017
Revised: January 10, 2018
Accepted: February 19, 2018
Published: March 29, 2018

REFERENCES

1. Schwarz, T.L. (2013). Mitochondrial trafficking in neurons. Cold Spring Harb. Perspect. Biol. *5*, a011304.
2. Sheng, Z.-H. (2017). The interplay of axonal energy homeostasis and mitochondrial trafficking and anchoring. Trends Cell Biol. *27*, 403–416.
3. Takihara, Y., Inatani, M., Eto, K., Inoue, T., Kreymerman, A., Miyake, S., Ueno, S., Nagaya, M., Nakanishi, A., Iwao, K., et al. (2015). *In vivo* imaging of axonal transport of mitochondria in the diseased and aged mammalian CNS. Proc. Natl. Acad. Sci. USA *112*, 10515–10520.
4. Milde, S., Adalbert, R., Elaman, M.H., and Coleman, M.P. (2015). Axonal transport declines with age in two distinct phases separated by a period of relative stability. Neurobiol. Aging *36*, 971–981.
5. Vagnoni, A., Hoffmann, P.C., and Bullock, S.L. (2016). Reducing Lissencephaly-1 levels augments mitochondrial transport and has a protective effect in adult *Drosophila* neurons. J. Cell Sci. *129*, 178–190.
6. Morsci, N.S., Hall, D.H., Driscoll, M., and Sheng, Z.-H. (2016). Age-related phasic patterns of mitochondrial maintenance in adult *Caenorhabditis elegans* neurons. J. Neurosci. *36*, 1373–1385.
7. Adalbert, R., and Coleman, M.P. (2013). Review: axon pathology in age-related neurodegenerative disorders. Neuropathol. Appl. Neurobiol. *39*, 90–108.
8. Fang, Y., Soares, L., Teng, X., Geary, M., and Bonini, N.M. (2012). A novel *Drosophila* model of nerve injury reveals an essential role of Nmnat in maintaining axonal integrity. Curr. Biol. *22*, 590–595.
9. Vagnoni, A., and Bullock, S.L. (2016). A simple method for imaging axonal transport in aging neurons using the adult *Drosophila* wing. Nat. Protoc. *11*, 1711–1723.
10. Li, L.B., Lei, H., Arey, R.N., Li, P., Liu, J., Murphy, C.T., Xu, X.Z., and Shen, K. (2016). The neuronal kinesin UNC-104/KIF1A is a key regulator of synaptic aging and insulin signaling-regulated memory. Curr. Biol. *26*, 605–615.
11. Tong, J.J., Schriner, S.E., McCleary, D., Day, B.J., and Wallace, D.C. (2007). Life extension through neurofibromin mitochondrial regulation and antioxidant therapy for neurofibromatosis-1 in *Drosophila melanogaster*. Nat. Genet. *39*, 476–485.
12. Wang, Z., Zhang, L., Liang, Y., Zhang, C., Xu, Z., Zhang, L., Fuji, R., Mu, W., Li, L., Jiang, J., et al. (2015). Cyclic AMP mimics the anti-ageing effects of calorie restriction by up-regulating Sirtuin. Sci. Rep. *5*, 12012.
13. Byers, D., Davis, R.L., and Kiger, J.A., Jr. (1981). Defect in cyclic AMP phosphodiesterase due to the dunce mutation of learning in *Drosophila melanogaster*. Nature *289*, 79–81.
14. Riera, C.E., and Dillin, A. (2015). Can aging be 'drugged'? Nat. Med. *21*, 1400–1405.
15. Avery, M.A., Rooney, T.M., Pandya, J.D., Wishart, T.M., Gillingwater, T.H., Geddes, J.W., Sullivan, P.G., and Freeman, M.R. (2012). WldS prevents axon degeneration through increased mitochondrial flux and enhanced mitochondrial Ca^{2+} buffering. Curr. Biol. *22*, 596–600.
16. Han, S.M., Baig, H.S., and Hammarlund, M. (2016). Mitochondria localize to injured axons to support regeneration. Neuron *92*, 1308–1323.
17. Cartoni, R., Norsworthy, M.W., Bei, F., Wang, C., Li, S., Zhang, Y., Gabel, C.V., Schwarz, T.L., and He, Z. (2016). The mammalian-specific protein Armcx1 regulates mitochondrial transport during axon regeneration. Neuron *92*, 1294–1307.
18. Hiruma, H., Saito, A., Kusakabe, T., Takenaka, T., and Kawakami, T. (2002). Neuropeptide Y inhibits axonal transport of particles in neurites of cultured adult mouse dorsal root ganglion cells. J. Physiol. *543*, 85–97.
19. Hwang, R.-D., Wiemerslage, L., LaBreck, C.J., Khan, M., Kannan, K., Wang, X., Zhu, X., Lee, D., and Fridell, Y.W. (2014). The neuroprotective effect of human uncoupling protein 2 (hUCP2) requires cAMP-dependent protein kinase in a toxin model of Parkinson's disease. Neurobiol. Dis. *69*, 180–191.
20. Ogawa, F., Murphy, L.C., Malavasi, E.L., O'Sullivan, S.T., Torrance, H.S., Porteous, D.J., and Millar, J.K. (2016). NDE1 and GSK3β associate with TRAK1 and regulate axonal mitochondrial motility: identification of cyclic AMP as a novel modulator of axonal mitochondrial trafficking. ACS Chem. Neurosci. *7*, 553–564.
21. Johnson, S.A., and Milner, M.J. (1987). The final stages of wing development in *Drosophila melanogaster*. Tissue Cell *19*, 505–513.
22. Raad, H., Ferveur, J.-F., Ledger, N., Capovilla, M., and Robichon, A. (2016). Functional gustatory role of chemoreceptors in *Drosophila* wings. Cell Rep. *15*, 1442–1454.
23. Ling, D., and Salvaterra, P.M. (2011). Robust RT-qPCR data normalization: validation and selection of internal reference genes during post-experimental data analysis. PLoS ONE *6*, e17762.
24. Pilling, A.D., Horiuchi, D., Lively, C.M., and Saxton, W.M. (2006). Kinesin-1 and dynein are the primary motors for fast transport of mitochondria in *Drosophila* motor axons. Mol. Biol. Cell *17*, 2057–2068.
25. Barkus, R.V., Klyachko, O., Horiuchi, D., Dickson, B.J., and Saxton, W.M. (2008). Identification of an axonal kinesin-3 motor for fast anterograde vesicle transport that facilitates retrograde transport of neuropeptides. Mol. Biol. Cell *19*, 274–283.
26. Jolly, A.L., and Gelfand, V.I. (2011). Bidirectional intracellular transport: utility and mechanism. Biochem. Soc. Trans. *39*, 1126–1130.
27. Puthanveettil, S.V., Monje, F.J., Miniaci, M.C., Choi, Y.B., Karl, K.A., Khandros, E., Gawinowicz, M.A., Sheetz, M.P., and Kandel, E.R. (2008). A new component in synaptic plasticity: upregulation of kinesin in the neurons of the gill-withdrawal reflex. Cell *135*, 960–973.

28. Mayr, B., and Montminy, M. (2001). Transcriptional regulation by the phosphorylation-dependent factor CREB. Nat. Rev. Mol. Cell Biol. *2*, 599–609.

29. Skalhegg, B.S., and Tasken, K. (2000). Specificity in the cAMP/PKA signaling pathway. Differential expression, regulation, and subcellular localization of subunits of PKA. Front. Biosci. *5*, D678–D693.

30. Impey, S., McCorkle, S.R., Cha-Molstad, H., Dwyer, J.M., Yochum, G.S., Boss, J.M., McWeeney, S., Dunn, J.J., Mandel, G., and Goodman, R.H. (2004). Defining the CREB regulon: a genome-wide analysis of transcription factor regulatory regions. Cell *119*, 1041–1054.

31. Zhang, X., Odom, D.T., Koo, S.H., Conkright, M.D., Canettieri, G., Best, J., Chen, H., Jenner, R., Herbolsheimer, E., Jacobsen, E., et al. (2005). Genome-wide analysis of cAMP-response element binding protein occupancy, phosphorylation, and target gene activation in human tissues. Proc. Natl. Acad. Sci. USA *102*, 4459–4464.

32. Okada, Y., Sato-Yoshitake, R., and Hirokawa, N. (1995). The activation of protein kinase A pathway selectively inhibits anterograde axonal transport of vesicles but not mitochondria transport or retrograde transport in vivo. J. Neurosci. *15*, 3053–3064.

33. Chang, C.-R., and Blackstone, C. (2007). Cyclic AMP-dependent protein kinase phosphorylation of Drp1 regulates its GTPase activity and mitochondrial morphology. J. Biol. Chem. *282*, 21583–21587.

34. Cribbs, J.T., and Strack, S. (2007). Reversible phosphorylation of Drp1 by cyclic AMP-dependent protein kinase and calcineurin regulates mitochondrial fission and cell death. EMBO Rep. *8*, 939–944.

35. Dagda, R.K., Gusdon, A.M., Pien, I., Strack, S., Green, S., Li, C., Van Houten, B., Cherra, S.J., III, and Chu, C.T. (2011). Mitochondrially localized PKA reverses mitochondrial pathology and dysfunction in a cellular model of Parkinson's disease. Cell Death Differ. *18*, 1914–1923.

36. Merrill, R.A., Dagda, R.K., Dickey, A.S., Cribbs, J.T., Green, S.H., Usachev, Y.M., and Strack, S. (2011). Mechanism of neuroprotective mitochondrial remodeling by PKA/AKAP1. PLoS Biol. *9*, e1000612.

37. Scherer, J., Yi, J., and Vallee, R.B. (2014). PKA-dependent dynein switching from lysosomes to adenovirus: a novel form of host-virus competition. J. Cell Biol. *205*, 163–177.

38. Hinckelmann, M.V., Zala, D., and Saudou, F. (2013). Releasing the brake: restoring fast axonal transport in neurodegenerative disorders. Trends Cell Biol. *23*, 634–643.

39. De Vos, K.J., and Hafezparast, M. (2017). Neurobiology of axonal transport defects in motor neuron diseases: opportunities for translational research? Neurobiol. Dis. *105*, 283–299.

40. Bilsland, L.G., Sahai, E., Kelly, G., Golding, M., Greensmith, L., and Schiavo, G. (2010). Deficits in axonal transport precede ALS symptoms *in vivo*. Proc. Natl. Acad. Sci. USA *107*, 20523–20528.

41. d'Ydewalle, C., Krishnan, J., Chiheb, D.M., Van Damme, P., Irobi, J., Kozikowski, A.P., Vanden Berghe, P., Timmerman, V., Robberecht, W., and Van Den Bosch, L. (2011). HDAC6 inhibitors reverse axonal loss in a mouse model of mutant HSPB1-induced Charcot-Marie-Tooth disease. Nat. Med. *17*, 968–974.

42. Kimura, N., Imamura, O., Ono, F., and Terao, K. (2007). Aging attenuates dynactin-dynein interaction: down-regulation of dynein causes accumulation of endogenous tau and amyloid precursor protein in human neuroblastoma cells. J. Neurosci. Res. *85*, 2909–2916.

43. Faits, M.C., Zhang, C., Soto, F., and Kerschensteiner, D. (2016). Dendritic mitochondria reach stable positions during circuit development. eLife *5*, e11583.

44. Lewis, T.L., Jr., Turi, G.F., Kwon, S.-K., Losonczy, A., and Polleux, F. (2016). Progressive decrease of mitochondrial motility during maturation of cortical axons *in vitro* and *in vivo*. Curr. Biol. *26*, 2602–2608.

45. Smit-Rigter, L., Rajendran, R., Silva, C.A.P., Spierenburg, L., Groeneweg, F., Ruimschotel, E.M., van Versendaal, D., van der Togt, C., Eysel, U.T., Heimel, J.A., et al. (2016). Mitochondrial dynamics in visual cortex are limited in vivo and not affected by axonal structural plasticity. Curr. Biol. *26*, 2609–2616.

46. Lane, M.E., and Kalderon, D. (1993). Genetic investigation of cAMP-dependent protein kinase function in *Drosophila* development. Genes Dev. *7*, 1229–1243.

47. Walker, J.A., Gouzi, J.Y., Long, J.B., Huang, S., Maher, R.C., Xia, H., Khalil, K., Ray, A., Van Vactor, D., Bernards, R., and Bernards, A. (2013). Genetic and functional studies implicate synaptic overgrowth and ring gland cAMP/PKA signaling defects in the *Drosophila melanogaster* neurofibromatosis-1 growth deficiency. PLoS Genet. *9*, e1003958.

48. Martin, M., Iyadurai, S.J., Gassman, A., Gindhart, J.G., Jr., Hays, T.S., and Saxton, W.M. (1999). Cytoplasmic dynein, the dynactin complex, and kinesin are interdependent and essential for fast axonal transport. Mol. Biol. Cell *10*, 3717–3728.

49. Sykiotis, G.P., and Bohmann, D. (2008). Keap1/Nrf2 signaling regulates oxidative stress tolerance and lifespan in *Drosophila*. Dev. Cell *14*, 76–85.

50. Yin, J.C., Wallach, J.S., Del Vecchio, M., Wilder, E.L., Zhou, H., Quinn, W.G., and Tully, T. (1994). Induction of a dominant negative CREB transgene specifically blocks long-term memory in *Drosophila*. Cell *79*, 49–58.

51. Nakamura, M., Baldwin, D., Hannaford, S., Palka, J., and Montell, C. (2002). Defective proboscis extension response (DPR), a member of the Ig superfamily required for the gustatory response to salt. J. Neurosci. *22*, 3463–3472.

52. Torroja, L., Chu, H., Kotovsky, I., and White, K. (1999). Neuronal overexpression of APPL, the *Drosophila* homologue of the amyloid precursor protein (APP), disrupts axonal transport. Curr. Biol. *9*, 489–492.

53. Rao, S., Lang, C., Levitan, E.S., and Deitcher, D.L. (2001). Visualization of neuropeptide expression, transport, and exocytosis in *Drosophila melanogaster*. J. Neurobiol. *49*, 159–172.

54. Hao, Y., Frey, E., Yoon, C., Wong, H., Nestorovski, D., Holzman, L.B., Giger, R.J., DiAntonio, A., and Collins, C. (2016). An evolutionarily conserved mechanism for cAMP elicited axonal regeneration involves direct activation of the dual leucine zipper kinase DLK. eLife *5*, e14048.

55. Godena, V.K., Brookes-Hocking, N., Moller, A., Shaw, G., Oswald, M., Sancho, R.M., Miller, C.C., Whitworth, A.J., and De Vos, K.J. (2014). Increasing microtubule acetylation rescues axonal transport and locomotor deficits caused by LRRK2 Roc-COR domain mutations. Nat. Commun. *5*, 5245.

STAR★METHODS

KEY RESOURCES TABLE

REAGENT or RESOURCE	SOURCE	IDENTIFIER
Antibodies		
rabbit anti-Kinesin-1 heavy chain	Cytoskeleton Inc.	Cat# AKIN01-B; RRID:AB_10707921
mouse anti-Complex-Vα	Thermo Fisher Scientific	Cat# 43-9800; RRID: AB_2533548
mouse anti-PKAc	Santa Cruz Biotechnology	Cat# sc-28315; RRID:AB_628136
rabbit anti-*Drosophila* PKAc (DC0)	Columbia University [46]	Produced and characterized by Daniel Kalderon
Chemicals, Peptides, and Recombinant Proteins		
8-Br-cAMP	Santa Cruz Biotechnology	Cat# sc-201564
PhosSTOP Phosphatase Inhibitor	Sigma-Aldrich	Cat# 4906837001
cOmplete Protease Inhibitor	Sigma-Aldrich	Cat# 11836170001
DTT	Sigma-Aldrich	Cat# 10197777001
LDS sample buffer	ThermoFisher	Cat# NP0008
SYPRO Ruby	Lonza	Cat# 50562
iScript Select cDNA synthesis kit	Bio-Rad	Cat# 1708896
EvaGreen Supermix	Bio-Rad	Cat# 1864034
Droplet Generator Oil	Bio-Rad	Cat# 1864005
RNase-free DNase Set	QIAGEN	Cat# 79254
Critical Commercial Assays		
cAMP Parameter Assay Kit	R&D Systems	Cat# KGE002B
RNeasy MINI kit	QIAGEN	Cat# 74104
Amersham ECL Prime Western Blotting Detection Reagent	GE-Healthcare	Cat# RPN2232
Amersham ECL Western Blotting Detection Kit	GE-Healthcare	Cat# RPN2108
Experimental Models: Organisms/Strains		
D. melanogaster: Oregon-R	Bloomington *Drosophila* Stock Center	Cat# 5; RRID:BDSC_5
D. melanogaster: w*; P{$w^{+mW.hs}$ = GawB}$dpr1^{PGaw}$, P{w^{+*} = UAS-GFP.U}2/CyO	Bloomington *Drosophila* Stock Center	Cat# 25083; RRID:BDSC_25083
D. melanogaster: w^{1118}; P{w^{+mC} = UAS-mito-HA-GFP.AP}2/CyO	Bloomington *Drosophila* Stock Center	BDSC Cat# 8442; RRID:BDSC_8442
D. melanogaster: w^{1118}; P{w^{+mC} = UAS-mito-HA-GFP.AP}3, e^{1}	Bloomington *Drosophila* Stock Center	BDSC Cat# 8443; RRID:BDSC_8443
D. melanogaster: P{w^{+m*} = Appl-GAL4.G1a}1, y^{1} w*	Bloomington *Drosophila* Stock Center	BDSC Cat# 32040; RRID:BDSC_32040
D. melanogaster: P{w^{+mC} = UAS-preproANF-EMD}136.3, y^{1} w*	Bloomington *Drosophila* Stock Center	BDSC Cat# 7001; RRID:BDSC_7001
D. melanogaster, RNAi of *luciferase*: y^{1} v^{1}; P{$y^{+t7.7}$ $v^{+t1.8}$ = TRiP.JF01355}attP2	Bloomington *Drosophila* Stock Center	BDSC Cat# 31603; RRID:BDSC_31603
D. melanogaster, RNAi of *dunce*: y^{1} v^{1}; P{$y^{+t7.7}$ $v^{+t1.8}$ = TRiP.JF02561}attP2	Bloomington *Drosophila* Stock Center	BDSC Cat# 27250; RRID:BDSC_27250
D. melanogaster, RNAi of *Pka-C1*: y^{1} v^{1}; P{$y^{+t7.7}$ $v^{+t1.8}$ = TRiP.JF01218}attP2	Bloomington *Drosophila* Stock Center	BDSC Cat# 31277; RRID:BDSC_31277
D. melanogaster, RNAi of *Pka-C1*: y^{1} v^{1}; P{$y^{+t7.7}$ $v^{+t1.8}$ = TRiP.JF01188}attP2	Bloomington *Drosophila* Stock Center	BDSC Cat# 31599; RRID:BDSC_31599
D. melanogaster: hs-PKA*	Harvard Medical School [47]	Produced and characterized by James Walker
D. melanogaster: P[Khc^{+}] 'PK9a'	UC Santa Cruz [48]	Produced and characterized by Bill Saxton
D. melanogaster: *gstD-GFP*	University of Rochester Medical Center [49]	Produced and characterized by Dirk Bohmann
D. melanogaster: *hs-dCREB2-b*	University of Wisconsin-Madison [50]	Produced and characterized by Jerry Yin

(*Continued on next page*)

Continued		
REAGENT or RESOURCE	SOURCE	IDENTIFIER
Oligonucleotides		
See Table S2 for the primers used for the amplification of target mRNAs by ddPCR.	This study	N/A
Software and Algorithms		
ImageJ	National Institute of Health, USA	https://imagej.nih.gov/ij/index.html
NIS-Elements	Nikon	https://www.nikoninstruments.com/en_GB/Products/Software
Quantasoft	Bio-Rad	http://www.bio-rad.com/en-us/product/qx200-droplet-digital-pcr-system
Excel	Microsoft	https://products.office.com/en-gb/excel
Prism	GraphPad	https://www.graphpad.com/scientific-software/prism/
Other		
Halocarbon oil (10S)	VWR	Cat# 24627.188
Glass microfiber filter papers	GE-Healthcare	Cat# 1822-021
X-ray films	GE-Healthcare	Cat# 28906837
DG8 cartridge	Bio-Rad	Cat# 1864008
Immobilon-P PVDF Transfer Membrane	Merck-Millipore	Cat# IPVH00010

CONTACT FOR REAGENT AND RESOURCE SHARING

Further information and requests for resources and reagents should be directed to and will be fulfilled by Alessio Vagnoni (alessio.vagnoni@kcl.ac.uk)

EXPERIMENTAL MODEL AND SUBJECT DETAILS

Drosophila strains and husbandry

The wild-type strain was Oregon-R. Strains containing the following transgenes were obtained from the Bloomington *Drosophila* Stock Center (Indiana University, USA): *dpr-Gal4* ([8, 51]; BL#25083), *UAS-mito::GFP* ([24]; BL#8442 or BL#8443); *appl-Gal4* ([52]; BL#32040); *UAS-preproANF::EMD* [53]; BL#7001); *UAS-luciferase-RNAi* (BL#31603); *UAS-dunce-RNAi* ([54]; BL#27250); *UAS-Pka-C1-RNAi* (BL#31277 - RNAi1; BL#31599 - RNAi2). The RNAi lines were generated by the Transgenic RNAi Project (TRiP) at Harvard Medical School, USA. *hs-PKA** flies [47] were provided by James Walker (Harvard University, USA). *P[Khc$^+$]* flies (‘PK9a’ stock [48]) were provided by Isabel Palacios (University of Cambridge, UK). The *gstD-GFP* reporter line [49] was provided by Sean Sweeney (University of York, UK). The *hs-dCREB2-b* flies [50] were provided by Mani Ramaswami (Trinity College Dublin, Ireland). Each transgene was present in one copy in the experimental genotypes. Within an experimental series, cohorts of flies were cultured using the same fly food. Flies were transferred to new food twice a week. Flies were cultured at 25°C unless stated otherwise using a 12-h-light–12-h-dark cycle. For experiments involving heat-shock, fly cultures were reared at 18°C throughout development and shifted to 30°C typically within 12 h of eclosion (for imaging young flies) or after 28 or 30 d (for 4-d or 24-h heat shock of aged flies, respectively).

METHOD DETAILS

Axonal transport assays

We recently published an in-depth protocol for the visualization and analysis of cargo transport in the wing nerve [9]. Briefly, flies that had been anaesthetised with CO_2 were immobilised, with wings outstretched, on a cover glass with a fine layer of Halocarbon oil (VWR). A second coverglass was then added on top of the fly to stabilize the sample. Wing nerves were imaged by spinning disk microscopy. Two spinning disk systems were used during the study, with the same system used for an entire experimental series: (1) PerkinElmer Ultraview ERS with a CSU21 scanning head (Yokogawa) and an inverted microscope stand (IX71 (Olympus)) equipped with a 60x PlanApo oil-immersion objective (1.4 NA) and a CCD camera (Orca ER (Hamamatsu)); (2) Nikon spinning disk system with a CSU-X1 scanning head (Yokogawa) and an inverted microscope stand (Eclipse Ti-E (Nikon)) equipped with a 60 x CFI Apo oil-immersion objective (1.4 NA) and an EM-CCD camera (Du 897 iXon Ultra (Andor)). Frame rates were 0.5/s and 1/s for mitochondria and DCVs, respectively. Image series were captured for 3 min. Tracking of mitochondria was performed manually (MTrackJ) on the raw movies by marking the start and end of each run. Tracking was performed blind to the experimental condition or genotype using the Randomizer macro (Tiago Ferreira, McGill University) in ImageJ. In cases where the sample shifted during filming, correction with

the StackReg plugin of ImageJ or the Alignment function of the NIS-Element software (Nikon) was performed before tracking. Movies with excessive focus drift were not quantified. The 'Advance Denoising' filter of the NIS-Elements software (Nikon) was applied to the time-lapse stacks before assembling the supplemental movies for display. We previously found no sex-related differences in mitochondrial transport [5]. Nonetheless, the same number of males and females were used in control and experimental conditions when analyzing mitochondrial transport. Only females were used for analysis of DCV movement as both the *appl-Gal4* and *UAS-ANF::EMD* transgenes are present on the X chromosome.

8-Br-cAMP feeding

For acute drug feeding prior to imaging, flies were treated essentially as described [55]. Newly eclosed flies or flies 28 d after eclosion were starved by removal from food for ~2 h at 25°C and placed on Whatman glass microfiber filter papers (GF/C grade, GE-Healthcare) soaked with 400 µL of a solution containing 10% sucrose alone (vehicle) or 10% sucrose and 100 µM 8-Br-cAMP (Santa Cruz Biotechnology). Filters were changed daily for 4 d before recording axonal transport in wing neurons. At the time of imaging, the age of the flies was 4 d after eclosion (young flies) and 32 d after eclosion (aged flies). For prolonged drug feeding, 8-Br-cAMP was added to standard fly food to a final concentration of 100 µM. Standard fly food was used in the vehicle control. Flies were transferred into fresh food (±8-Br-cAMP) twice a week until imaging.

Immunoblotting and protein staining

Protein extracts from wings and heads were prepared following a protocol adapted from Vagnoni et al., 2016 [5]. Wings and heads were collected by cutting at the wing root close to the thorax of the animal or at the base of the head, respectively, with fine spring scissors. Typically, 100 wings or 20 heads were collected in a 1.5 mL Eppendorf tube kept on dry ice. Tissues were collected by brief centrifugation at 16,100 x *g* and ground on ice with a plastic hand-held pestle before adding 300 µL (for heads) or 200 µL (for wings) of lysis buffer (50 mM Tris pH 7.4, 150 mM NaCl, 5 mM EDTA, 1x PhosSTOP Phosphatase Inhibitor (Roche) and 1x cOmplete Protease Inhibitor (Roche)). After addition of Triton X-100 to a final concentration of 1%, the samples were further homogenized by multiple passages through a 23G syringe needle. Lysates were spun at 16,100 x *g* for 30 min at 4°C. 40 mM DTT was added to the collected supernatant, mixed with LDS sample buffer (Novex-Life Technologies) and boiled for 10 min at 90°C before electrophoresis. After gel electrophoresis and protein transfer, membranes were incubated with the following primary antibodies: rabbit anti-Kinesin-1 heavy chain (AKIN01, Cytoskeleton Inc., diluted 1:1000), mouse anti-Complex-Vα (MitoSciences-Life Technologies, clone 15H4C4, diluted 1:5000 for wings and 1:10,000 for heads), mouse anti-PKAc (Santa Cruz Biotechnology, clone A2, diluted 1:1000) or rabbit anti-*Drosophila* PKAc (DC0) ([46]; diluted 1:1000 (a gift from Daniel Kalderon (Columbia University, USA)). After washing with PBS containing 0.1% Tween, HRP-conjugated secondary antibodies and chemiluminescent substrate (ECL or ECL Prime detection systems (GE-Healthcare)) were used to reveal the protein-derived signal on X-ray films (GE-Healthcare). Typically, between 2% and 10% of total lysate sample was loaded per gel lane. Background corrected band intensities were quantified using the Gel Analyzer tool in ImageJ. Signals from the target protein were normalized to the mean signal for the control sample or young fly sample on the same film. For the data in Figures 3B, 3D, and 4A, signals from the target protein were additionally normalized for the Complex Vα signal for the same sample. For staining with SYPRO Ruby (Lonza), gels were fixed in 50% methanol/7% acetic acid for 1 h following electrophoresis. Overnight incubation in SYPRO Ruby gel stain was followed by washing in 10% methanol/7% acetic acid for 40 min before imaging with a ChemiDoc MP imaging system (Bio-Rad).

Assessment of cAMP levels

cAMP abundance in *Drosophila* wings was assayed with the cAMP Parameter Assay Kit (R&D Systems). 100 wings were homogenized in 600 µL of Cell Lysis Buffer from the kit and the lysate centrifuged twice at 600 x *g* for 5 min at 4°C to remove cellular debris. After collecting the supernatant, the assay was carried out immediately according to the manufacturer's instructions. The absorbance was measured at 450 nm with a PHERAstar FS microplate reader (BMG Labtech) with correction wavelength set at 570 nm. The final cAMP concentration per mL of extract was calculated by interpolating the absorbance values on a cAMP standard curve.

RT-ddPCR

Wings were collected as described above. RNA was isolated with a RNeasy MINI kit (QIAGEN) from 140 wings per condition, and DNase treatment performed 'on-column' with RNase-free DNase Set (QIAGEN) according to the manufacturer's instruction. RT reactions were carried out at 42°C for 80 min with Oligo(dT)$_{20}$ and the iScript Select cDNA synthesis kit (Bio-Rad). 10 ng purified RNA was added to each reaction. Prior to ddPCR, end-point PCR was used to check the quality of the RT reactions and verify the presence of the mRNA of interest. ddPCR was performed with the QX200 Droplet Digital PCR System (Bio-Rad), following the manufacturer's instructions. 1 µL of RT reaction was mixed with the EvaGreen Supermix (Bio-Rad) and forward and reverse primers (Table S2) in a final volume of 20 µl. The reaction mix was loaded into a DG8 cartridge (Bio-Rad) and 70 µL of Droplet Generator Oil (Bio-Rad) was added before droplet production in the QX200 Droplet Generator (Bio-Rad). 40 µL of droplets was then used for each ddPCR replicate with the cycling conditions recommended by the manufacturer, except the annealing/extension temperature was set at 59°C. The ddPCR output was read in a QX200 Droplet Reader (Bio-Rad) and the results visualized with QuantaSoft software (Bio-Rad). For each reaction, data from > 15,000 droplets were analyzed, and partitioned into two clearly distinguishable populations of positive droplets (where target amplification took place) and negative droplets (no target amplification).

Oxidative stress and focal protein accumulation assays

Flies containing the *gstD-GFP* transgene were mounted in an imaging chamber for spinning disk confocal microscopy, as described above. Z stacks of neuronal areas were acquired in multiple positions along the wing nerve. The different genotypes within a single experiment were imaged with the same laser power and camera gain on the same day. Reporter fluorescent intensity was measured from cell bodies using Z-projections of 5 focal planes. The average pixel fluorescence intensity of each region of interest was then calculated in ImageJ after subtraction of the background fluorescence signals from each image. The 'Smooth' filter of ImageJ was applied to images for presentation purposes. Flies overexpressing the *UAS-GFP* transgene present in the *dpr-GAL4* stock were used to quantify the amount of wing nerve affected by focal protein accumulation in the fifth week after eclosion, as described previously [5]. Briefly, the wing nerve was divided into 8 equal segments, with a segment scored as positive if at least one focal accumulation of GFP was present.

QUANTIFICATION AND STATISTICAL ANALYSIS

Statistical analysis and data plotting was performed using Excel (Microsoft) and Prism (GraphPad). Details of the quantification methods for the different experiments are provided under each heading in the Method Details section. Details of statistical evaluations are provided in the figure legends, and the numbers of samples indicated in the figures. Non-parametric tests were used when the sample size was $\leq$ 10. Multiple comparison corrections were applied when more than two groups were compared.

Current Biology, Volume *28*

Supplemental Information

A cAMP/PKA/Kinesin-1 Axis Promotes the Axonal Transport of Mitochondria in Aging *Drosophila* Neurons

Alessio Vagnoni and Simon L. Bullock

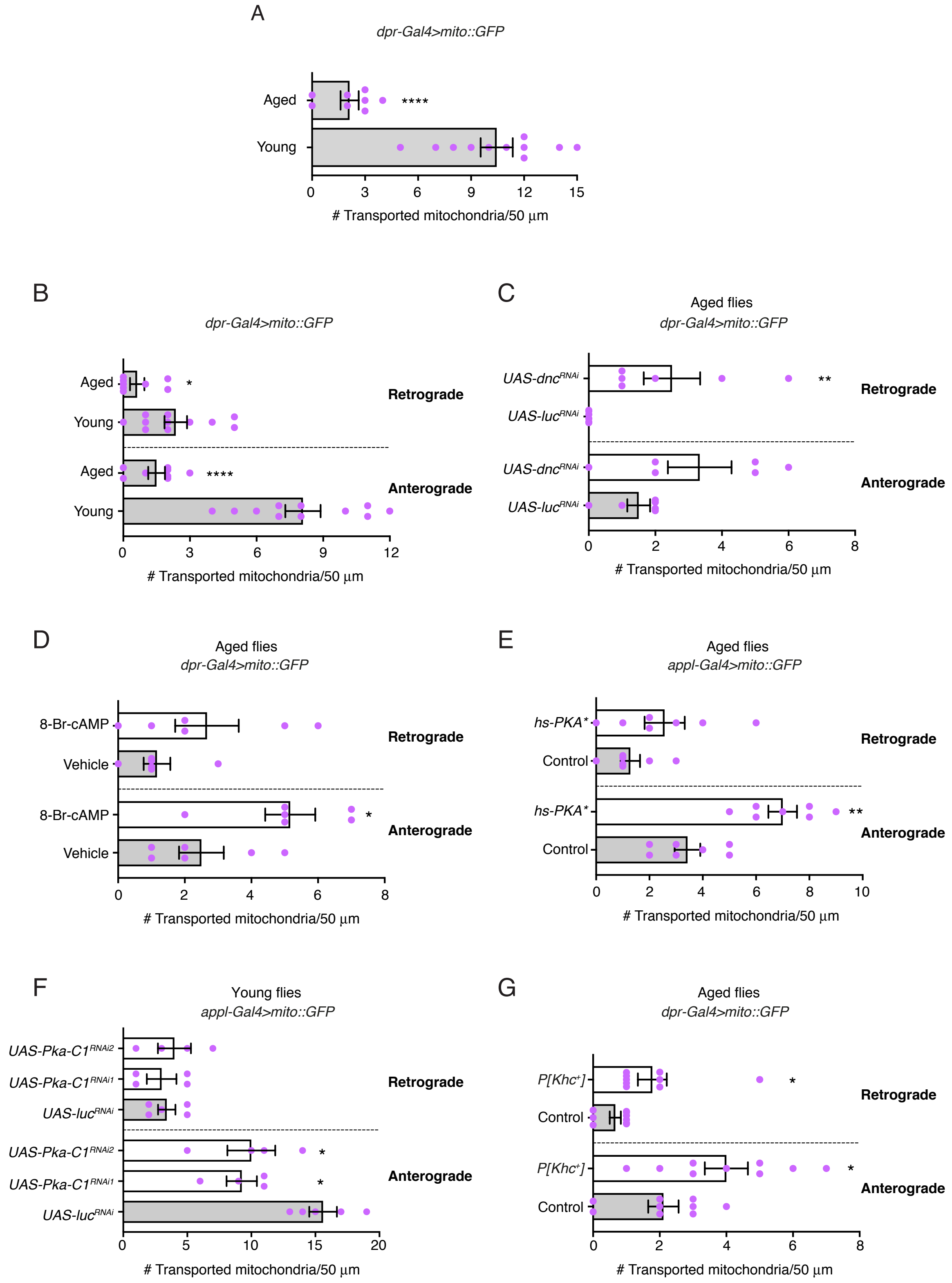

A
dpr-Gal4>mito::GFP
Aged
Young
****
Transported mitochondria/50 µm
B
dpr-Gal4>mito::GFP
Aged
Young
Retrograde
Anterograde
*
****
Transported mitochondria/50 µm
C
Aged flies
dpr-Gal4>mito::GFP
UAS-dnc^RNAi
UAS-luc^RNAi
Retrograde
Anterograde
**
Transported mitochondria/50 µm
D
Aged flies
dpr-Gal4>mito::GFP
8-Br-cAMP
Vehicle
Retrograde
Anterograde
*
Transported mitochondria/50 µm
E
Aged flies
appl-Gal4>mito::GFP
hs-PKA*
Control
Retrograde
Anterograde
**
Transported mitochondria/50 µm
F
Young flies
appl-Gal4>mito::GFP
UAS-Pka-C1^RNAi2
UAS-Pka-C1^RNAi1
UAS-luc^RNAi
Retrograde
Anterograde
*
Transported mitochondria/50 µm
G
Aged flies
dpr-Gal4>mito::GFP
P[Khc+]
Control
Retrograde
Anterograde
*
Transported mitochondria/50 µm

Figure S1. Supplemental data for mitochondrial movements in wing neurons. Related to Figures 1, 2 and 4.

(A) Example of magnitude of reduction in mitochondrial transport in wing neurons of aged flies vs young flies. In these and other panels, mitochondria were marked with mito::GFP expressed in wing neurons using the Gal4-UAS system.

(B-G) Charts show number of transported mitochondria in the retrograde and anterograde direction per 50 μm of axonal tract per 3 min of image aquisition. Data in A and B derive from the same movies. Young and aged flies in A and B were, respectively, 2 days and 30 days after eclosion when imaged. Note that in F only anterograde motion of mitochondria was significantly reduced by RNAi of *Pka-C1*. Despite the specific decrease in anterograde transport, the total number of mitochondria in the axonal segment was not altered (Figure 2E). This finding is consistent with previous evidence that net transport is not the sole determinant of the steady-state distribution of mitochondria in axons [S1]. Statistical significance was evaluated with a Mann-Whitney U-test (A-E and G) and a one-way ANOVA with Dunnett's multiple comparisons correction (F). *P*-value: *, <0.05; **, <0.01; ****, <0.0001. Magenta circles are values for individual wings; error bars are SEM.

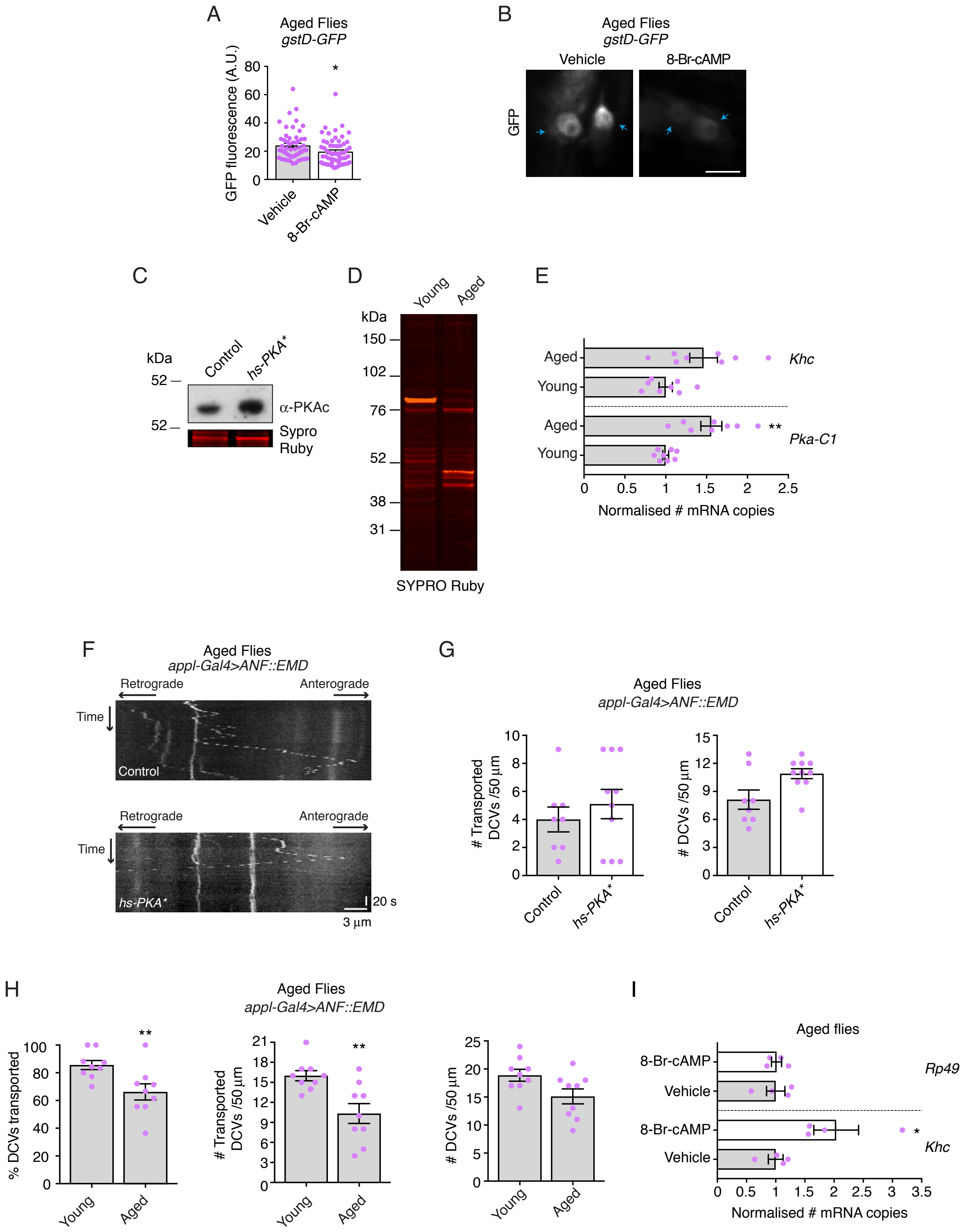
A
Aged Flies
gstD-GFP
GFP fluorescence (A.U.)
80
60
40
20
0
*
Vehicle
8-Br-cAMP
B
Aged Flies
gstD-GFP
Vehicle
8-Br-cAMP
GFP
C
kDa
Control
hs-PKA*
52
α-PKAc
52
Sypro Ruby
D
Young
Aged
kDa
150
102
76
52
38
31
SYPRO Ruby
E
Aged
Young
Aged
Young
Khc
**
Pka-C1
0
0.5
1
1.5
2
2.5
Normalised # mRNA copies
F
Aged Flies
appl-Gal4>ANF::EMD
Retrograde
Anterograde
Time
Control
Retrograde
Anterograde
Time
hs-PKA*
20 s
3 μm
G
Aged Flies
appl-Gal4>ANF::EMD
Transported DCVs /50 μm
10
8
6
4
2
0
Control
hs-PKA*
DCVs /50 μm
15
12
9
6
3
0
Control
hs-PKA*
H
Aged Flies
appl-Gal4>ANF::EMD
% DCVs transported
100
80
60
40
20
0
**
Young
Aged
Transported DCVs /50 μm
21
18
15
12
9
6
3
0
**
Young
Aged
DCVs /50 μm
25
20
15
10
5
0
Young
Aged
I
Aged flies
8-Br-cAMP
Vehicle
Rp49
8-Br-cAMP
Vehicle
*
Khc
0
0.5
1
1.5
2
2.5
3
3.5
Normalised # mRNA copies

Figure S2. Supplemental data on cAMP/PKA pathway activation. Related to Figures 1, 2 and 3.
(A) GFP fluorescence in cell bodies of wing neurons of 30-day-old *gstD-GFP* flies fed throughout adulthood with food supplemented with 8-Br-cAMP or standard food (vehicle). GFP is expressed in response to oxidative stress through activation of the *gstD* enhancer elements by the Nrf2 transcription factor [S2, S3].
(B) Images showing examples of GFP signals in cell bodies (blue arrows) of wing neurons expressing *gstD-GFP*. Scale bar: 5 µm.
(C) Top panel: Immunoblot for PKA* using extracts of heads of 4-day-old control (wild-type) or *hs-PKA** flies cultured at 30°C for 4 days. The more intense band in the *hs-PKA** sample confirms expression of PKA* from the transgene. Note that the antibody recognises both the endogenous *Drosophila* PKAc and exogenous mouse PKA*. Bottom panel shows a segment of SYPRO Ruby-stained gel from the same samples, which contains an abundant protein that is not responsive to PKA* induction.
(D) SYPRO Ruby-stained gel of wing extracts of young (day 2) and aged (day 30) wild-type flies.
(E) Abundance of *Pka-C1* and *Khc* mRNAs in young (2 day) and aged (day 30) wild-type flies. mRNA level was assessed by RT-ddPCR, with values normalised to those for the vehicle control. Magenta circles are values for individual technical replicates from two independent reverse transcription reactions. There was a statistically significant increase in *Pka-C1* mRNA in the aged sample compared to the young sample (1.5-fold difference), which could conceivably reflect a mechanism to compensate for reduced PKAc protein.
(F) Kymographs illustrating axonal transport of DCVs in wing neurons of heat-shocked control and *hs-PKA** aged flies.
(G) Quantification of the number of transported and total DCVs in the axonal tract in the wing of heat-shocked control and *hs-PKA** aged flies. There was a modest, but not statistically significant, increase in the total number of DCVs in axons when PKA* was overexpressed.
(H) Quantification of DCV transport in the axonal tract in the wing of young (day 2) and aged (day 30) animals. There was a statistically significant reduction in the percentage of DCVs that are transported in the aged flies compared to the young flies. We previously monitored DCV motility in wild-type wing neurons over the first 16 days of adult life and found no decline in transport [S4]; this contrasts with the sharp decline in mitochondrial transport observed during this period [S4].
In G and H, magenta circles are values for individual wings, with each wing filmed for 3 minutes.
(I) Abundance of *Khc* and *Rp49* mRNA in wings of aged wild-type flies (day 31) fed either vehicle or 8-Br-cAMP for the preceding 24 hours. mRNA abundance was assessed by RT-ddPCR, with values normalised to those for the vehicle control.
Statistical significance was evaluated with the Mann-Whitney U-test. *P*-values: *, <0.05; **, <0.01; error bars are SEM.

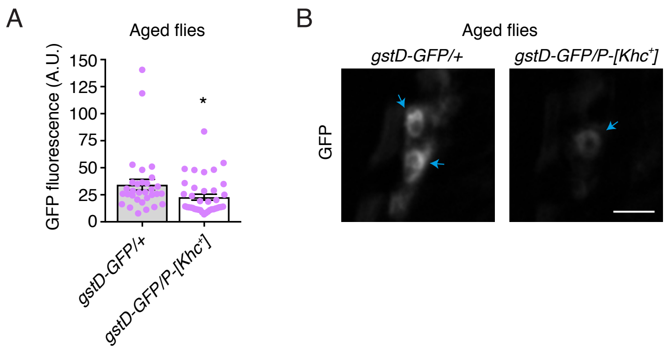

Figure S3. Supplemental data on Khc overexpression. Related to Figure 4.
(A) Quantification of GFP fluorescence in cell bodies of wing neurons of 30-day-old *gstD-GFP/+* or *gstD-GFP/P[Khc*$^{+}$*]* flies. Magenta circles are values for individual Z-projections from a total of 10 wings of each genotype. Statistical significance was evaluated with a two-tailed Student's *t*-test. *P*-values: *, <0.05; error bars are SEM.
(B) Images showing examples of GFP signals in cell bodies (arrows) of wing neurons expressing *gstD-GFP*. Scale bar: 5 µm.

	Mean velocity (µm s^{-1})		Mean run length (µm)	
Condition	Anterograde	Retrograde	Anterograde	Retrograde
**Related to Fig. 1**				
Aged flies:				
luc^{RNAi}	0.19 ± 0.04 (9)	NA (0)	7.49 ± 1.93 (9)	NA (0)
dnc^{RNAi}	0.17 ± 0.02 (20)	0.33 ± 0.04 (15)	12.00 ± 1.71 (20)	10.80 ± 1.48 (15)
Young flies:				
Vehicle	0.20 ± 0.01 (37)	0.32 ± 0.03 (24)	14.21 ± 1.74 (37)	11.00 ± 1.57 (24)
8-Br-cAMP	0.20 ± 0.01 (47)	0.33 ± 0.04 (17)	10.97 ± 1.17 (47)	11.65 ± 2.71 (17)
Aged flies:				
Vehicle	0.23 ± 0.02 (15)	0.47 ± 0.05 (7)	16.96 ± 2.99 (15)	7.99 ± 2.64 (7)
8-Br-cAMP	0.24 ± 0.01 (31)	0.35 ± 0.06 (16)	15.79 ± 1.51 (31)	10.21 ± 1.56 (16)
**Related to Fig. 2**				
Young flies:				
Control	0.19 ± 0.01 (40)	0.33 ± 0.05 (20)	16.66 ± 1.3 (40)	10.93 ± 1.56 (20)
*hs-PKA**	0.25 ± 0.01 (52) **	0.31 ± 0.04 (19)	16.90 ± 1.35 (52)	14.90 ± 2.95 (19)
Aged flies:				
Control	0.22 ± 0.02 (24)	0.29 ± 0.06 (9)	12.14 ± 1.95 (24)	11.88 ± 2.99 (9)
*hs-PKA**	0.23 ± 0.02 (49)	0.38 ± 0.05 (18)	10.91 ± 1.02 (49)	13.45 ± 2.07 (18)
Young flies:				
luc^{RNAi}	0.21 ± 0.01 (78)	0.39 ± 0.05 (17)	12.25 ± 1.01 (78)	12.19 ± 2.55 (17)
$Pka\text{-}C1^{RNAi1}$	0.28 ± 0.02 (37) ***	0.37 ± 0.06 (12)	12.71 ± 1.31 (37)	13.39 ± 2.47 (12)
$Pka\text{-}C1^{RNAi2}$	0.29 ± 0.01 (40) ***	0.55 ± 0.07 (16)	12.18 ± 1.10 (40)	8.60 ± 1.21 (16)
**Related to Fig. 4**				
Young flies:				
Control	0.18 ± 0.01 (104)	0.33 ± 0.03 (45)	12.96 ± 0.84 (104)	11.39 ± 0.87 (45)
$P[Khc^{+}]$	0.21 ± 0.07 (110) *	0.37 ± 0.03 (59)	12.41 ± 0.87 (110)	13.81 ± 1.31 (59)
Aged flies:				
Control	0.20 ± 0.02 (19)	0.23 ± 0.06 (6)	13.56 ± 2.00 (19)	8.12 ± 2.42 (6)
$P[Khc^{+}]$	0.19 ± 0.01 (36)	0.35 ± 0.05 (16)	12.45 ± 1.39 (36)	9.07 ± 1.91 (16)

Table S1. **Velocity and run length of motile mitochondria after manipulation of the cAMP/PKA pathway or Khc overexpression. Related to Figures 1, 2 and 4.**

Number of runs are in parentheses.

NA: not applicable as no retrograde movements were observed.

For comparisons involving two groups, statistical significance was evaluated with a Mann-Whitney U test (when $n < 10$) or two-tailed Student's t-test (when $n > 10$). An ANOVA test with Dunnett's multiple comparison correction was used for *Pka-C1* RNAi experiments, which involved three groups. *P*-value: *, <0.05; **, <0.01; ***, <0.0001; errors are SEM.

Both RNAi treatment and overexpression of PKAc significantly increased anterograde velocity in young flies, suggesting complex regulation of this parameter at this stage.

Mean run lengths were not significantly affected by changes in Khc concentration, consistent with travel distances of certain cargoes being insensitive to changes in the number of associated kinesin-1 motors [S5, S6].

Name	Sequence
Khc (FWD)	CAGAACATCATCCTCACCAAC
Khc (RVS)	CCTCGCTCTCCTCGTTTAC
Rp49 (FWD)	CGGAAACTCAATGGATACTGC
Rp49 (RVS)	CGACTGGTGGCGGATGAAGTG
Rap2L (FWD)	ACTTCCGTGCATTACGTGCG
Rap2L (RVS)	CCGACCCGAGCACAACAACT
14-3-3 (FWD)	CATGAACGATCTGCCACCAAC
14-3-3 (RVS)	CTCTTCGCTCAGTGTATCCAAC
eIF-1A (FWD)	ATCAGCTCCGAGGATGACGC
eIF-1A (RVS)	GCCGAGACAGACGTTCCAGA
Milton (FWD)	CAGGATCAGCTGAAGCAACA
Milton (RVS)	AGCTCACAGTGCAGTGAATCC
Miro (FWD)	ACTCATCTAATCCCCGTTCC
Miro (RVS)	GCAGCAACTTGTACTTGTCAC
Pka-C1 (FWD)	GGATCAGCCGATTCAGATCTAC
Pka-C1 (RVS)	AATATCATTGACGCCCGCC
Sgg (FWD)	CTGGTAACAACAGTAGCAGC
Sgg (RVS)	CATTAGTCGCGCCTATAGC

Table S2. **Details of gene-specific primers used for RT-ddPCR. Related to Figures 3, S2 and Key Resources Table.**

Supplemental References

S1. Lopez-Domenech, G., Higgs, N.F., Vaccaro, V., Ros, H., Arancibia-Carcamo, I.L., MacAskill, A.F., and Kittler, J.T. (2016). Loss of dendritic complexity precedes neurodegeneration in a mouse model with disrupted mitochondrial distribution in mature dendrites. Cell Rep. 17, 317-327.

S2. Sykiotis, G.P., and Bohmann, D. (2008). Keap1/Nrf2 signaling regulates oxidative stress tolerance and lifespan in *Drosophila*. Dev. Cell 14, 76-85.

S3. Milton, V.J., Jarrett, H.E., Gowers, K., Chalak, S., Briggs, L., Robinson, I.M., and Sweeney, S.T. (2011). Oxidative stress induces overgrowth of the *Drosophila* neuromuscular junction. Proc. Natl. Acad. Sci. USA 108, 17521-17526.

S4. Vagnoni, A., Hoffmann, P.C., and Bullock, S.L. (2016). Reducing Lissencephaly-1 levels augments mitochondrial transport and has a protective effect in adult *Drosophila* neurons. J. Cell Sci. 129, 178-190.

S5. Shubeita, G.T., Tran, S.L., Xu, J., Vershinin, M., Cermelli, S., Cotton, S.L., Welte, M.A., and Gross, S.P. (2008). Consequences of motor copy number on the intracellular transport of Kinesin-1-driven lipid droplets. Cell 135, 1098-1107.

S6. Jamison, D.K., Driver, J.W., Rogers, A.R., Constantinou, P.E., and Diehl, M.R. (2010). Two kinesins transport cargo primarily via the action of one motor: implications for intracellular transport. Biophys. J. 99, 2967-2977.
